# Evolutionary Genomics of Peach and Almond Domestication

**DOI:** 10.1534/g3.116.032672

**Published:** 2016-10-04

**Authors:** Dianne Velasco, Josh Hough, Mallikarjuna Aradhya, Jeffrey Ross-Ibarra

**Affiliations:** * Department of Plant Sciences, University of California, Davis, California 95616; † United States Department of Agriculture, Agricultural Research Service, National Clonal Germplasm Repository, Davis, California 95616; ‡ Center for Population Biology, University of California, Davis, California 95616; § Genome Center, University of California, Davis, California 95616

**Keywords:** *Prunus persica*, peach, *Prunus dulcis*, almond, domestication, mating system

## Abstract

The domesticated almond [*Prunus dulcis* (L.) Batsch] and peach [*P. persica* (Mill.) D. A. Webb] originated on opposite sides of Asia and were independently domesticated ∼5000 yr ago. While interfertile, they possess alternate mating systems and differ in a number of morphological and physiological traits. Here, we evaluated patterns of genome-wide diversity in both almond and peach to better understand the impacts of mating system, adaptation, and domestication on the evolution of these taxa. Almond has around seven times the genetic diversity of peach, and high genome-wide $F_{ST}$ values support their status as separate species. We estimated a divergence time of ∼8 MYA (million years ago), coinciding with an active period of uplift in the northeast Tibetan Plateau and subsequent Asian climate change. We see no evidence of a bottleneck during domestication of either species, but identify a number of regions showing signatures of selection during domestication and a significant overlap in candidate regions between peach and almond. While we expected gene expression in fruit to overlap with candidate selected regions, instead we find enrichment for loci highly differentiated between the species, consistent with recent fossil evidence suggesting fruit divergence long preceded domestication. Taken together, this study tells us how closely related tree species evolve and are domesticated, the impact of these events on their genomes, and the utility of genomic information for long-lived species. Further exploration of this data will contribute to the genetic knowledge of these species and provide information regarding targets of selection for breeding application, and further the understanding of evolution in these species.

*Prunus* is a large genus in the family Rosaceae with ∼200 species, including multiple domesticated crops such as almond, apricot, cherry, peach, and plum (Rehder 1940; Potter 2011). Peach [*P. persica* (Mill.) D. A. Webb] and almond [*P. dulcis* (L.) Batsch] are two of the three most economically important domesticates in *Prunus* globally, and share a number of similarities, including perenniality, precocity, and genome size and organization (Baird *et al.* 1994; Arús *et al.* 2012). However, the two species also have striking differences. While peaches are harvested for their indehiscent fleshy mesocarp, almonds are harvested for their seed, encased in a stony endocarp and a leathery, dehiscent mesocarp and exocarp (see Supplemental Material, File S1 and Figure S1). And while almond, like most *Prunus* species, exhibits *S*-RNase-based gametophytic self-incompatibility, peach is self-compatible (Hedrick *et al.* 1917; Wellington *et al.* 1929). Almond and peach also differ for other traits, such as life span (Gradziel 2011), chilling requirements (Alonso *et al.* 2005; Dozier *et al.* 1990; Scorza and Okie 1991), and adventitious root generation (Kester and Sartori 1966).

Domestication of almond and peach occurred independently ∼5000 yr ago in the Fertile Crescent and China (Zohary *et al.* 2012), respectively, followed by global dissemination beginning before 1300 BCE (Hedrick *et al.* 1917; Edwards 1975; Gradziel 2011; Zheng *et al.* 2014). The few obvious domestication traits in almond are reduced toxicity, thinner endocarp, and increased seed size, while domestication in peach is characterized by diverse fruit morphology (size, color, texture, shape, etc.) and self-compatibility. Other traits not typically associated with domestication, such as precocity, adventitious rooting, graft compatibility, or tree architecture, may also have been selected during domestication or subsequent breeding (reviewed in Miller and Gross 2011; Spiegel-Roy 1986). Efforts to identify the wild progenitors of either almond or peach by examining species relationships within subgenus *Amygdalus* have produced inconsistent species trees and numerous polytomies (Mowrey *et al.* 1990; Browicz and Zohary 1996; Ladizinsky 1999; Aradhya *et al.* 2004; Bassi and Monet 2008; Zeinalabedini *et al.* 2010; Verde *et al.* 2013). Given uncertainty in the wild progenitors and the difficulties associated with long generation times, QTL-mapping approaches to investigate peach or almond domestication are thus impractical. In contrast, comparatively fast and inexpensive sequencing makes population genetic approaches (*cf*. Ross-Ibarra *et al.* 2007) an attractive option, enabling the identification of domestication loci and study of the genome-wide impacts of changes in mating system.

Both domestication and mating system have been shown to shape genomic patterns of diversity in annual species (Glémin *et al.* 2006; Doebley *et al.* 2006; Hazzouri *et al.* 2013; Slotte *et al.* 2013), but the impacts of these forces on tree species remain poorly documented (McKey *et al.* 2010; Miller and Gross 2011; Gaut *et al.* 2015; but see Hamrick *et al.* 1992 for relevant analyses of allozyme diversity data). Mating system differences between closely related species pairs has been shown to significantly affect many aspects of genome evolution in *Arabidopsis*, *Capsella*, and *Collinsia*, including lower nucleotide diversity, higher linkage disequilibrium (LD), and reduced effective population size (*N_e_*) (Hazzouri *et al.* 2013; Slotte *et al.* 2013; Wright *et al.* 2013). Demographic bottlenecks associated with domestication may also reduce diversity genome-wide, and selection during domestication will reduce diversity even further at specific loci (Glémin *et al.* 2006; Doebley *et al.* 2006). While studies in perennials, particularly tree fruit crops, suggest they have lost little genetic diversity due to domestication (reviewed in Miller and Gross 2011), recent analysis of resequenced peach genomes are consistent with lower genetic diversity and higher LD across the genome compared to related wild species (Verde *et al.* 2013; Cao *et al.* 2014). No such genome-wide analysis of diversity in almonds currently exists, however, and little is known about how differences in mating system affect changes in diversity during domestication.

Here, we leverage both new and published genome sequences to present an evolutionary genomic analysis of the effects of domestication and mating system on diversity in both almond and peach. Understanding the impact of mating system will expand the basic knowledge of genome evolution in a perennial species pair with contrasting mating systems, and identification of candidate domestication loci will provide an opportunity to assess convergence during domestication and compare tree domestication to that of annual crops.

## Materials and Methods

### Samples

We used 13 almond and 13 peach genomes for all analyses, which included both public and newly resequenced data (Table 1 and Table S1). In addition, we used one peach-almond *F*_1_ hybrid and one peach with Nonpareil almond in its pedigree as checks for admixture analysis. For this study, we resequenced nine almonds, one peach, an almond-peach *F*_1_ hybrid, and the plum *P. cerasifera* as an outgroup (Table 1 and Table S1). Fresh leaves and dormant cuttings collected from multiple sources were either desiccated with silica or stored at 4° prior to DNA isolation. We isolated DNA following a modified CTAB method (Doyle 1987).

**Table 1:** *P*. *dulcis*, *P. persica*, and outgroup species used in analyses

Species	*n*	Average Depth	Reference
*P. dulcis*	4	7.76	Koepke *et al.* (2013)
*P. dulcis*	9	19.34	This study
*P. persica*	10	19.13	Verde *et al.* (2013)
*P. persica*	2	13.78	Ahmad *et al.* (2011)
*P. persica*	1	37.36	This study
*P. cerasifera*	1	35.02	This study

Libraries for eight of the almond samples were prepared at UC Davis. We quantified the sample DNA with Quanti-iT Picogreen dsDNA assay (Invitrogen, Life Technologies) and then fragmented 1 μg with a Bioruptor (Diagenode) for 11 cycles of 30 sec ON and 30 sec OFF per cycle. The resulting DNA fragment ends were then repaired with NEBNext End Repair (New England BioLabs) followed by the addition of deoxyadenosine triphosphate to the 3’ ends with Klenow Fragment (New England BioLabs). We then ligated barcoded Illumina TrueSeq adapters (Affymetrix) to the A-tailed fragments with Quick Ligase (New England BioLabs). Between each enzymatic step, we washed the DNA with Sera-Mag SpeedBeads (GE Life Sciences, Pittsburgh). The resulting libraries were quantified with a Qubit (Life Technologies) and sized using a BioAnalyzer DNA 12,000 chip (Agilent Technologies). Libraries were sent to UC Berkeley (Berkeley, Qb3) for quantification by qPCR, multiplexing, and sequencing for 100 bp paired-end reads in a single HiSeq 2000 (Illumina) lane. DNA from the remaining samples (Table 1 and Table S1) was submitted to BGI (Shenzen, China) for library preparation and sequenced using 100 bp paired-end reads at their facility in Hong Kong. Sequence data are available from SRA (http://www.ncbi.nlm.nih.gov/sra) and the associated run numbers are given in Table S1.

### Analysis

#### Quality control and mapping:

All FASTQ files were trimmed of remnant adapter sequences using Scythe (github.com/vsbuffalo/scythe) and then further trimmed for base quality with Sickle (github.com/najoshi/sickle) using default parameters for both. Trimmed reads were then aligned to the *P. persica* v1.0 reference (www.rosaceae.org) using BWA-MEM (Li 2013) with a minimum seed length of 10 and internal seed length of 2.85. After filtering for a minimum mapping quality of 30 and base quality of 20, sequence depth averaged 15.8× (4.7× to 34.6×) in almond and 19.7× (11.2× to 35.4× in peach; Figure S2, Table S1).

#### Diversity and candidate gene identification:

We estimated inbreeding coefficients using *ngsF* in the *ngsTools* suite (Fumagalli *et al.* 2014), and then calculated genotype likelihoods in ANGSD (Korneliussen *et al.* 2014) incorporating our inbreeding estimates. We calculated several population genetics statistics, including pairwise nucleotide diversity ($\theta_{\pi };$ Nei and Li 1979), Tajima’s *D* (*D*; Tajima 1989), Fay and Wu’s *H* (*H*; Fay and Wu 2000), and Zeng’s *E* (*E*; Zeng *et al.* 2006) using the *thetaStat* subprogram in ANGSD. Diversity values were estimated in overlapping 1000 bp windows with 50 bp steps, removing windows with < 150 bp of sequence after filtering. Additionally, we calculated a normalized $\theta_{\pi }$ value by dividing per window $\theta_{\pi }$ by mean $\theta_{\pi }$ in each species. To identify candidate genes possibly selected during domestication, we filtered for genes in the lowest 5% empirical quantile of each diversity statistic. We further analyzed candidate loci for gene ontology (GO) using *P. persica* protein gene identifiers in the singular enrichment analysis tool and Fisher’s exact test using default statistical options at the AgriGO website (http://bioinfo.cau.edu.cn/agriGO/).

#### Population comparisons:

We treated peach samples and almond samples as two populations to evaluate population structure. We performed a principal component analysis (PCA) with *ngsPopGen* (Fumagalli *et al.* 2014), and used *NGSadmix* (Skotte *et al.* 2013) to perform an admixture analysis and assign proportions of almond and peach population to individuals using *K* = 2 through *K* = 6 as the number of potential subpopulations. Finally, we examined population differentiation by estimating $F_{ST}$ genome-wide and in sliding windows (1000 bp windows with a 50 bp step) after removing windows with <150 bp of sequence.

#### Estimating historical changes in N_e_:

To model the history of these species and infer the historical changes in effective population size that may have occurred prior to or during domestication, we analyzed peach and almond samples using the Multiple Sequentially Markovian Coalescent (MSMC) method (Schiffels and Durbin 2014). This approach uses the observed pattern of mutations in multiple individuals to infer the time to the most recent common ancestor between pairs of sampled alleles, and provides maximum-likelihood estimation of population size as a function of time. Using the msmc software (github.com/stschiff/msmc) and msmc-tools (github.com/stschiff/msmc-tools), we applied this method to 10 individuals from our study (five peach and five almond samples; peach: PP02, PP03, PP04, PP05, and PP13; almond: PD03, PD04, PD05, PD06, and PD07) in two separate analyses. For each individual, we first identified SNPs for each chromosome using samtools mpileup (v. 1.3.1) with a minimum mapping and base quality cut off of 20. We filtered sites for depth < 15 using VCFtools (v. 0.1.13), and removed indels using bcftools (v. 1.3.1). To estimate population size changes during the recent past (since domestication), we ran the full MSMC model for peach and almond separately using the combined set of five samples for each run. To estimate changes in $N_{e}$ over a longer time period (2 MYA), we applied the PSMC’ model (see Schiffels and Durbin 2014) to each sample individually. To convert the mutation-scaled coalescent times and population sizes obtained from these analyses, we divided by a mutation rate of $\mu =10^{-8}$ mutations per nucleotide per generation, and assumed a generation time of 10 yr for both peach and almond. The models and inference algorithms for PSMC’ and MSMC are available from github.com/stschiff/msmc, and our code for analyzing peach and almond samples is available from https://github.com/houghjosh/peach.

#### Gene expression:

We downloaded 10 SRA RNA-seq runs from four peach and almond tissues (Table S2). All runs were from either general transcriptome sequencing (Jo *et al.* 2015) or controls of differential expression experiments (Wang *et al.* 2013; Mousavi *et al.* 2014; Sanhueza *et al.* 2015). We then converted the runs into their paired FASTQ files using SRA-toolkit (v. 2.3.4) and quantified expression for each run separately against the peach transcriptome (v. 1.0) using kallisto (Bray *et al.* 2016). For each sequencing run, kallisto outputs the transcripts per million (TPM), a within library proportional measurement, for each gene. Each gene was then annotated with its candidate or noncandidate status based on $F_{ST},$ $\theta_{\pi },$ Tajima’s *D*, Zeng’s *E*, or Fay and Wu’s *H* for both almond and peach. We also calculated the number of tissues in which each gene was expressed and the mean expression level in each tissue (across runs in which the gene was expressed).

### Data availability

The authors state that all data necessary for confirming the conclusions presented in the article are represented fully within the article.

## Results and Discussion

### Diversity

Genome-wide nucleotide diversity ($\theta_{\pi };$ Figure S5 and Figure S6) in almond is nearly sevenfold higher than in peach (0.0186 and 0.0027, respectively), and these differences were more pronounced in nongenic regions of the genome (Table 2 and Table S4). Though differences in diversity between peach and almond have been known from analyses using multiple marker systems (Mowrey *et al.* 1990; Byrne 1990; Martínez-Gómez *et al.* 2003; Aradhya *et al.* 2004), this study is the first comparison of whole genome sequences using multiple diverse individuals from both species. Previous genome scans of peach found low levels of genetic diversity compared to the closely related wild species, *P. kansuensis*, *P. mira*, and *P. davidiana* (Verde *et al.* 2013; Cao *et al.* 2014). Of these, only *P. davidiana* is outcrossing, and Verde *et al.* (2013) found it had the greatest nucleotide diversity of the species they examined, ∼threefold higher than domesticated peach. Despite its domesticated status, almond retains more genetic diversity than any of the peach species studied thus far, suggesting that mating system explains more of the differences in diversity among species than domestication. Finally, we observed considerable variation in diversity statistics among chromosomes in both species, including up to twofold differences in nucleotide diversity in peach (Table S4), perhaps suggesting the relatively recent effects of selection during domestication.

**Table 2:** Genome-wide, genic, and nongenic diversity statistics and neutrality test values

Species	Sites	*$\theta_{\pi }\times 10^{3}$*	*D*	*H*	*E*
Almond	Genome	18.37	−1.15	−0.12	−0.22
	Genic	10.57	−1.49	−0.03	−0.35
	Nongenic	25.67	−0.83	−0.20	−0.10
Peach	Genome	2.70	−0.49	−0.56	0.14
	Genic	1.67	−0.51	−0.50	0.10
	Nongenic	3.61	−0.47	−0.62	0.17

*D*, Tajima’s *D*; *H*, Fay and Wu’s *H*; *E*, Zeng’s *E*.

Mean values of Tajima’s *D* were negative for both almond and peach (Table 2), suggesting that a genome-wide excess of rare variants likely consistent with a history of population expansion. Strongly negative values of Tajima’s *D* have recently been reported in *Populus tremuloides*, a species also inferred to have undergone postglacial population expansion in the Quaternary Wang *et al.* (2016). While the wild progenitors of almond and peach are not definitively known, the current range of wild almond species is much larger than that of wild peach taxa, perhaps reflecting either contrasting initial population sizes or differential expansion of ancestral progenitors during interglacial periods following the Last Glacial Maximum (20 kbp; LGM).

### Historical changes in N_e_

To investigate the demographic factors that may have contributed to the strong allele frequency skews that we observed in both peach and almond (Table 2), we conducted a whole-genome analysis of coalescent rates between haplotypes through time using MSMC (Schiffels and Durbin 2014). The results from this analysis provide the first detailed comparisons of demography in both peach and almond, and enabled us to obtain estimates of population size changes from ∼2 MYA up to ≈1000 yr ago (*i.e.*, the last 100 generations; Figure S8). We found no evidence for a domestication-associated population bottleneck in either peach or almond (Figure S8A). Instead, our results suggest that almond experienced a population expansion following a bottleneck ≈20,000 yr ago, consistent with our observations of a strongly negative Tajima’s *D* and perhaps due to rapid human-mediated dispersal from east Asia (Delplancke *et al.* 2012). In peach, our results suggest a gradual decline in $N_{e}$ beginning ≈2 MYA (Figure S8B), and extending to 5000 yr ago, after which the effective population size remains very low. Although our results do not support a bottleneck in peach, the gradual decline in $N_{e}$ starting in the distant past (≈2 MYA; Figure S8B) is consistent with the low overall diversity we observe (Table 2), and may reflect a shift to a higher selfing rate (Charlesworth 2003).

Overall, our analyses suggest that, although population bottlenecks or extreme population expansions have occurred during domestication in many crop species (Meyer *et al.* 2012; Beissinger *et al.* 2016), neither peach nor almond appear to have experienced such events. In this respect, our results mirror those from other domesticated woody perennial crop species, including grape and apple, which are also reported to lack domestication bottlenecks but maintain much of their ancestral genetic diversity (Myles *et al.* 2011; Gross *et al.* 2014). This difference between annual and perennial domesticated crops may be due to the unique life cycle features of perennials, including a long generation time, overlapping generations, and a typically outcrossing mating system, as well as a more recent period of domestication (Gaut *et al.* 2015). That we also found a large reduction in $N_{e}$ and neutral diversity in peach despite no evidence for a population bottleneck also highlights the possibility that, within woody perennials, mating system differences may play an important role in determining the propensity of these species to have domestication-associated bottlenecks.

### Inbreeding

We estimated the average inbreeding coefficient (*F*) for almond and peach to be 0.002 (0.000–0.027) and 0.197 (0.000–0.737), respectively (Table S3 and Figure S3). Although two self-compatible almond varieties are included in this study, none of our almond samples are derived from self-fertilization, supporting the low estimated inbreeding values. Peaches in general are self-compatible (with the exception of male-sterile varieties), and three of the peach varieties sampled (PP06, PP08, and PP15) have inbreeding values consistent with self-pollination in the preceding generation (*F* = 0.74, 0.53, and 0.56, respectively). Consistent with its known history as the result of open-pollination (Hedrick *et al.* 1917), the Georgia Belle peach variety sampled was estimated to have F=0.

While the estimated inbreeding value for almond is not unexpected given that it is self-incompatible, the average for peach is lower than previously estimated selfing rates (*s*) of 0.5−0.86 (F=0.33−0.75 from F=(s/2−s); Fogle and Dermen 1969; Fogle 1977; Miller *et al.* 1989; Akagi *et al.* 2016). While the widely cited Miller *et al.* (1989) estimate was based on a single isozyme marker and is thus unable to separate self-fertilization with outcrossing to close relatives, the Akagi *et al.* (2016) estimate based on 5180 SNP markers is also high (s=0.50−0.68 from F=0.33−0.52). Our estimates are much closer to those from Aranzana *et al.* (2002), who estimated s=0.148 (F=0.08) from 35 microsatellites. In addition to differences in marker systems, these discrepancies are likely due at least in part to sampling, with estimates from outcrossed pedigrees (Aranzana *et al.* 2002) lower than those from landraces (Akagi *et al.* 2016). Broad examination of pedigree records, however, suggests our estimate of inbreeding is likely reasonable, as more than 67% of the 600 peaches in Okie (1998) were the result of outcrossing (Aranzana *et al.* 2002), including several of the varieties sampled here (Hedrick *et al.* 1917).

### Population structure

**Figure 1:**
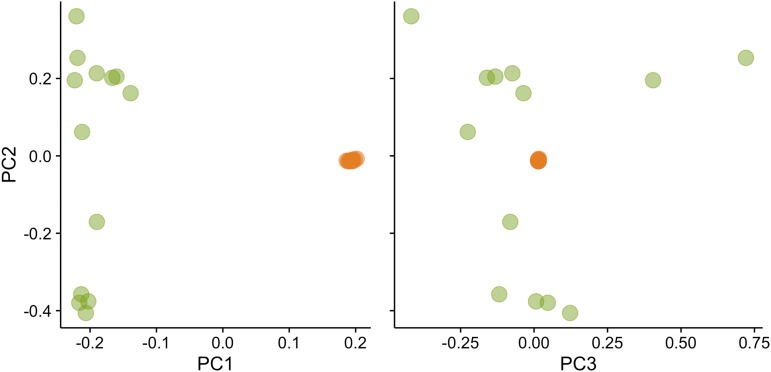
Principle component (PC) analysis of almond (green) and peach (orange).

Genome-wide, our data are consistent with previous estimates (Aradhya *et al.* 2004) in finding strong genetic differentiation between almond and peach (weighted $F_{ST}=0.605,$ Figure S7 and Table S4). Like $F_{ST},$ PCA also clearly distinguished almond from peach samples, primarily along PC1 (Figure 1). However, while PC2 and PC3 provided no further separation of peach samples, they do allow further separation of almond samples (Figure 1).

**Figure 2:**
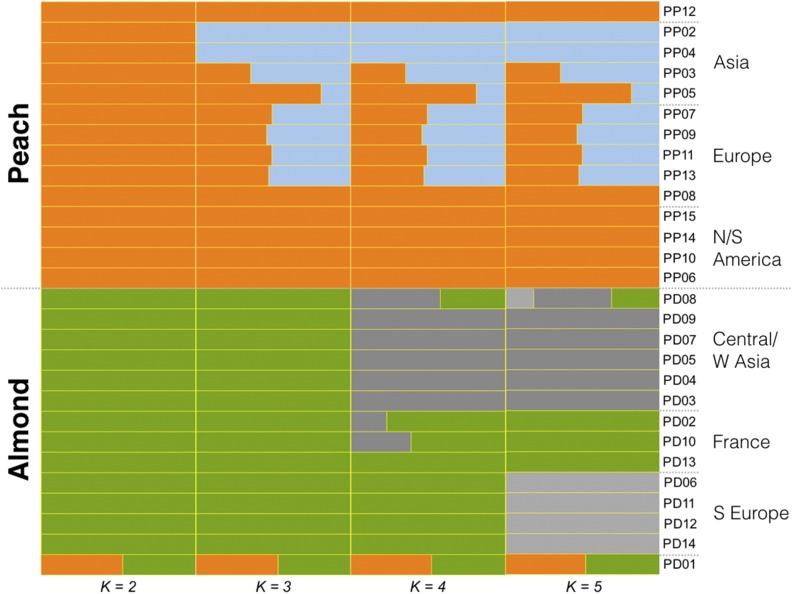
Admixture proportion of almond (PD) and peach (PP) for *K* = 2 through *K* = 5. With the exception of the purported hybrids, PD01 and PP12, sample origins generally correspond with an east (top) to west (bottom) orientation for each type (Table S1).

Admixture analysis clearly assigns individuals to either almond or peach populations at *K* = 2 (green and orange, respectively), including the correct identification of PD01 as an almond-peach F1 hybrid (Figure 2). Peach sample PP12, in contrast, should show ∼12.5% almond based on its pedigree (Fresnedo-Ramírez *et al.* 2013) but in this analysis does not differ from other New World peaches in its assigned proportions. The fact that PP12 shows fewer total variants than PP13 (“Georgia Belle”; Fresnedo-Ramírez *et al.* 2013) is also inconsistent with recent almond ancestry, suggesting possible errors in the recorded pedigree.

Increasing the number of clusters (*K*), we find evidence for population substructure in both peach and almond (Figure 2 and Figure S4) distinguished by geographic origin or breeding status. In the admixture plot (Figure 2), within both almond and peach groups, samples at the top have more eastern origins (Central Asia or China, respectively), whereas those toward the bottom have more western origins (Spain or New World, respectively). Almond samples from China, Pakistan, Iran, and Turkey (PD09, PD07, PD05, PD04, and PD03) group together at both *K* = 4 and *K* = 5. At *K* = 5, a Mediterranean group of Italian and Spanish samples (PD06, PD11, PD12, and PD14) is identified, perhaps reflecting gene flow from North Africa into Spain and Italy (Delplancke *et al.* 2013). At *K* = 6, PD01 forms a unique cluster and several other almonds shift assignments, suggesting an overestimation of the number of subgroups (Figure S4). Similar overall patterns of structure in peach and almond were found in previous studies (Li *et al.* 2013; Micheletti *et al.* 2015; Shen *et al.* 2015; Delplancke *et al.* 2013) as well, suggesting the use of local varieties as founders, limited exchange between Asian and European breeding programs, or recent utilization of diverse genetic resources is not reflected in the sampling. The foundations of most modern almond breeding programs began within the past century, due in part to the challenges of understanding self-incompatibility, whereas the self-compatible peach has had more widespread efforts directed toward its development for millenia (though western breeding increased or intensified only within the past 10–20 generations).

All of our analyses of differentiation provide unequivocal evidence distinguishing almonds from peaches, strongly supporting their status as distinct species. Previous molecular analyses have estimated a broad range of divergence times between these species, from 2.5 MYA (Vieira *et al.* 2008) to more than 47 MYA (Chin *et al.* 2014). One compelling idea for the origin of peach and almond is that climatic changes after Himalayan orogeny and Tibetan Plateau uplift led to isolation of an hypothesized ancestral species resulting in allopatric divergence of peach from almond (Chin *et al.* 2014). Consistent with this possibility, our estimates of $F_{ST}$ and nucleotide diversity give a divergence time of ≈8 MY under a simple model of divergence in isolation (*cf*. Holsinger and Weir 2009), assuming a mutation rate of $\mu =10^{-8}$ per nucleotide and generation time of ≈10 yr. This corresponds to a period of climatic change following significant geologic activity and uplift specifically in the northeastern section of the Tibetan Plateau (Fang *et al.* 2007; Molnar *et al.* 2010).

### Candidate loci

We next scanned the genomes of both almond and peach for potential candidate genes targeted by selection during domestication. In the lowest 5% quantile of Zeng’s *E*, we found 1334 and 1315 genes in peach and almond, respectively. Of these, peach and almond share 104, nearly double that expected by chance (permutation p-value <0.001) and suggesting convergence in the process of domestication. In almond, candidate genes showed enrichment for GO categories related to protein amino acid phosphorylation, ATP biosynthetic processes, regulation of ADP ribosylation factor (ARF) protein signal transduction, membrane and nucleus cellular components, ATP binding, ATPase and protein tyrosine kinase activities, and zinc ion binding; candidate genes in peach showed enrichment for the GO category related to cellular catabolic processes. We also identified the 1314 genes showing the greatest differentiation between species (top 5% quantile of $F_{ST}$), but while these genes were enriched for a number of GO categories (Table S5) no clear patterns emerged.

**Figure 3:**
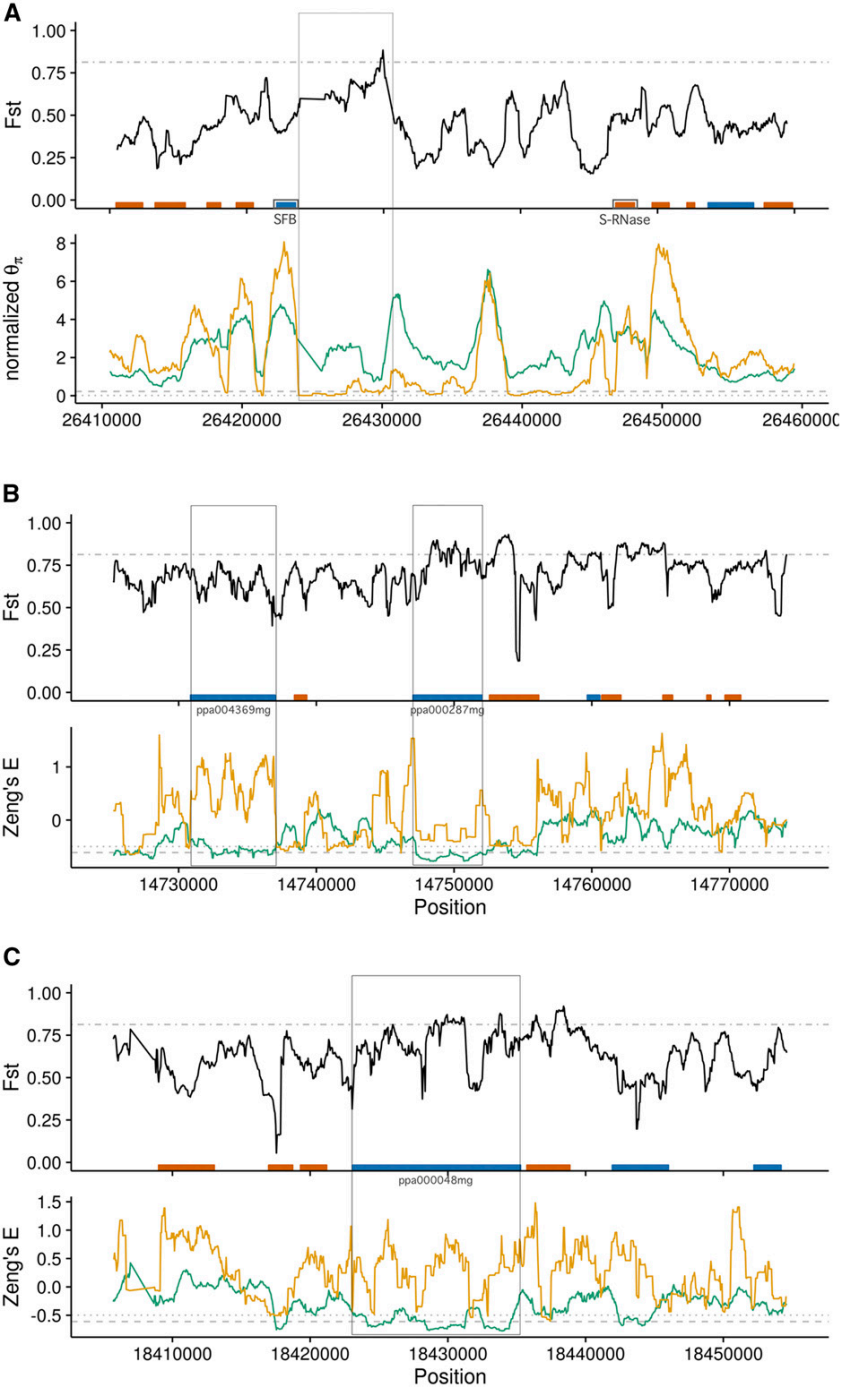
Select 50 kb windows of the genome with high divergence ($F_{ST}$) and either low normalized $\theta_{\pi }$ (A) or Zeng’s *E* (B and C) of almond (green) and peach (orange). Genes annotated in the peach reference genome are represented in the $F_{ST}$ plot by boxes colored by their location on the plus strand (blue) or minus strand (red). In the $F_{ST}$ plots, the gray lines indicate the upper 5% threshold, whereas in the $\theta_{\pi }$ and Zeng’s *E* panels the gray lines indicate the lower 5% thresholds of almond (dashed) and peach (dotted). Regions of interest, as described in the text, are boxed across adjacent panels and genes labeled. (A) *S*-locus divergence and diversity with *S*-locus genes, *SFB* (blue), and *S*-RNase (red), located on opposite sides of the central gap. Diversity in peach is drastically reduced immediately 3′ to *SFB* but only somewhat reduced 3′ to *S*-RNase, as might be expected for a linked locus. (B) and (C) Loci of interest on chromosome 3.

We first investigated the *S*-locus in order to examine a genomic region known to differ between almond and peach both in sequence and function (Tao *et al.* 2007; Hanada *et al.* 2014). The *S*-locus controls gametophytic self-incompatibility in *Prunus* (reviewed in Wu *et al.* 2013). The *S*-locus haplotype block consists of two genes, *S*-RNase and the *S*-haplotype-specific F-box (*SFB*), which function in the pistil and pollen, respectively. In our data, the intergenic region 3′ to both the *S*-RNase and *SFB* loci shows elevated differentiation with one extremely high peak and low nucleotide diversity in peach (Figure 3A), observations consistent with recent work showing peach having only five known *S*-haplotypes, two of which have identical *SFB* alleles (Tao *et al.* 2007; Hanada *et al.* 2014).

Windows in the lowest 5% quantile of the summary statistics investigated were generally enriched for genic regions of the genome in both taxa, but the signal in peach was weak and enrichment was not consistent across all statistics evaluated (Table S6). Nonetheless, a number of individual regions genome-wide showed strong signatures of selection. We examined 50 kb regions with contiguous windows in the bottom 5% quantile to focus our investigations of candidate genes. We focused on regions in both species for which there were overlapping regions of high $F_{ST}$ and low $\theta_{\pi }$ or Zeng’s *E*, as these were significant for both peach and almond (permutation p-values 0−0.034; Table 3).

**Table 3:** Permutation probability for the overlap of neutrality test or $\theta_{\pi }$ selected candidate genes with high $F_{ST}$ selected candidate genes

Species	Tajima’s *D*	Fay and Wu’s *H*	Zeng’s *E*	$\theta_{\pi }$
Almond	0	0	0	0
Peach	0.5854	0.3336	0.0342	0

While many intergenic and putative regulatory regions also showed interesting patterns in diversity statistics, we examined two regions of chromosome 3 with moderate to high $F_{ST}$ and divergent values of Zeng’s *E* between peach and almond, specifically low values of Zeng’s *E* in almond (Figure 3, B and C). The first of these regions (Figure 3B), contains the uncharacterized genes ppa004369mg (position 3:14730867..14736998; Uniprot identifier M5WRK6_PRUPE) and ppa00287mg (position 3:14747030..14752018; Uniprot identifier M5WX95_PRUPE), which have similarity to γ-aminobutyrate (GABA) transaminases in *Malus domesticus* and Myosin-1 in *Gossypium arboreum*, respectively. GABA is involved in signaling and nuclear regulation of cell wall modification and cell death through repression and activation, respectively, while GABA transaminases degrade GABA in the mitochondria and are reported to have a role in pollen–pistil interactions. Myosins are cellular motor proteins that act in concert with actin filaments for intracellular transport and cellular structure. The second region of interest on chromosome 3 (Figure 3C) contains the uncharacterized gene ppa000048mg (position 3: 18423078..18435264, Uniprot identifier M5XGZ7_PRUPE). This gene is in the GO category of protein *N*-linked glycosylation, and though it has high protein BLAST similarity among many species, few were annotated. Further investigation of additional regions with limited homology to characterized genes or functional information may be warranted given the poor characterization of genes in these species.

Given the importance of fruit morphology in peach, we hypothesized that selection during domestication and subsequent breeding may have targeted genes primarily expressed in fruit tissue. To test this hypothesis, we compared gene expression in four tissues (peach fruit and leaf, and almond ovary and anther) to candidate gene status. Candidates were overrepresented among genes expressed in all tissues, and we saw no evidence of enrichment for tissue-specific expression in any of the four tissues ($\chi^{2}$ test showed significant underenrichment in most cases; Table S7). Even among genes showing tissue-specific expression, we found no difference in expression between domestication candidates and noncandidates. We did, however, find that genes showing strong differentiation between almond and peach (highest 5% tail of $F_{ST}$) showed higher levels of expression in both leaves and fruit. While we have no clear *a priori* hypothesis predicting differences in leaf-specific expression, higher fruit-specific expression among $F_{ST}$ is certainly of note given the striking differences in fruit morphology between the species.

Contrary to our predictions, we find no evidence that domestication candidates are enriched for genes showing unusual patterns or levels of expression. Recent results, however, suggest that larger fruits may have much predated domestication. Seeds of a 2.6 MY-old fossil peach, *P. kunmingensis*, were recently reported to be nearly identical to modern peaches (Su *et al.* 2015), and the observed correlation between seed size and fruit size in peach (Zheng *et al.* 2014) suggests that fruit size was likely larger as well. Our finding that fruit-specific genes showing the strongest differentiation between species are more highly expressed is, thus, at least consistent with the possibility of selection for differences in fruit morphology between peach and almond predating domestication.

### Conclusions

One of the primary questions regarding the domestication of perennial crops, particularly tree crops, is its genetic basis (Miller and Gross 2011). Here, we have examined two closely related domesticated tree species with alternate mating systems in an attempt to tease apart the genomic signatures of domestication and mating system, and better understand these processes in perennial species. In addition to presenting evidence consistent with mating system effects in determining overall patterns of genetic diversity, our results identify numerous genes and genomic regions showing evidence of selection, and provide evidence of convergence in the domestication of almond and peach, and that fruit was not preferentially targeted during domestication but likely selected much earlier during species divergence. Finally, the high-coverage sequence we provide for a number of important cultivars may be useful to breeders and geneticists in identifying the causal basis of quantitative trait loci or developing marker sets for marker-assisted selection or genomic prediction.

## Supplementary Material

Supplemental Material

## References

[bib1] Ahmad R. Parfitt D. E. Fass J. Ogundiwin E. Dhingra A., 2011 Whole genome sequencing of peach (*Prunus persica* L.) for SNP identification and selection. BMC Genomics 12: 569. 22108025 10.1186/1471-2164-12-569 PMC3253712

[bib2] Akagi T. Hanada T. Yaegaki H. Gradziel T. M. Tao R., 2016 Genome-wide view of genetic diversity reveals paths of selection and cultivar differentiation in peach domestication. DNA Res. 23: 271–282. 27085183 10.1093/dnares/dsw014 PMC4909313

[bib3] Alonso J. Ansón J. Espiau M. R. Socias i Company, 2005 Determination of endodormancy break in almond flower buds by a correlation model using the average temperature of different day intervals and its application to the estimation of chill and heat requirements and blooming date. J. Am. Soc. Hortic. Sci. 130: 308–318.

[bib4] Aradhya M. K. Weeks C. Simon C. J., 2004 Molecular characterization of variability and relationships among seven cultivated and selected wild species of *Prunus* L. using amplified fragment length polymorphism. Sci. Hortic. (Amsterdam) 103: 131–144.

[bib5] Aranzana M. Garcia-Mas J. Carbo J. Arús P., 2002 Development and variability analysis of microsatellite markers in peach. Plant Breed. 121: 87–92.

[bib6] Arús P. Verde I. Sosinski B. Zhebentyayeva T. Abbott A. G., 2012 The peach genome. Tree Genet. Genomes 8: 531–547.

[bib7] Baird W. V. Estager A. S. Wells J. K., 1994 Estimating nuclear DNA content in peach and related diploid species using laser flow cytometry and DNA hybridization. J. Am. Soc. Hortic. Sci. 119: 1312–1316.

[bib8] Bassi D. Monet R., 2008 Botany and taxonomy, pp. 1–36 in *The Peach: Botany*, *Production and Uses*, chap 1., edited by Layne D. R. Bassi D. CABI, Wallingford, UK.

[bib9] Beissinger T. M. Wang L. Crosby K. Durvasula A. Hufford M. B., 2016 Recent demography drives changes in linked selection across the maize genome. Nature Plants 2: 16084. 27294617 10.1038/nplants.2016.84

[bib10] Bray N. L. Pimentel H. Melsted P. Pachter L., 2016 Near-optimal probabilistic RNA-seq quantification. Nat. Biotechnol. 34: 525–527. 27043002 10.1038/nbt.3519

[bib11] Browicz K. Zohary D., 1996 The genus *Amygdalus* L. (Rosaceae): species relationships, distribution and evolution under domestication. Genet. Resour. Crop Evol. 43: 229–247.

[bib12] Byrne D., 1990 Isozyme variability in four diploid stone fruits compared with other woody perennial plants. J. Hered. 81: 68–71.

[bib13] Cao K. Zheng Z. Wang L. Liu X. Zhu G., 2014 Comparative population genomics reveals the domestication history of the peach, *Prunus persica*, and human influences on perennial fruit crops. Genome Biol. 15: 415. 25079967 10.1186/s13059-014-0415-1 PMC4174323

[bib14] Charlesworth D., 2003 Effects of inbreeding on the genetic diversity of populations. Philos. Trans. R. Soc. Lond. B Biol. Sci. 358: 1051–1070. 12831472 10.1098/rstb.2003.1296 PMC1693193

[bib15] Chin S.-W. Shaw J. Haberle R. Wen J. Potter D., 2014 Diversification of almonds, peaches, plums and cherries–molecular systematics and biogeographic history of *Prunus* (Rosaceae). Mol. Phylogenet. Evol. 76: 34–48. 24631854 10.1016/j.ympev.2014.02.024

[bib16] Delplancke M. Alvarez N. Espíndola A. Joly H. Benoit L., 2012 Gene flow among wild and domesticated almond species: insights from chloroplast and nuclear markers. Evol. Appl. 5: 317–329. 25568053 10.1111/j.1752-4571.2011.00223.x PMC3353361

[bib17] Delplancke M. Alvarez N. Benoit L. Espíndola A. Joly H. I., 2013 Evolutionary history of almond tree domestication in the mediterranean basin. Mol. Ecol. 22: 1092–1104. 23189975 10.1111/mec.12129

[bib18] Doebley J. F. Gaut B. S. Smith B. D., 2006 The molecular genetics of crop domestication. Cell 127: 1309–1321. 17190597 10.1016/j.cell.2006.12.006

[bib19] Doyle J. J., 1987 A rapid DNA isolation procedure for small quantities of fresh leaf tissue. Phytochem. Bull. 19: 11–15.

[bib20] Dozier W. Powell A. Caylor A. McDaniel N. Carden E., 1990 Hydrogen cyanamide induces budbreak of peaches and nectarines following inadequate chilling. HortScience 25: 1573–1575.

[bib21] Edwards, S., 1975 The almond industry of Mexico. Master’s Thesis, Oregon State University, Corvallis.

[bib22] Fang X. Zhang W. Meng Q. Gao J. Wang X., 2007 High-resolution magnetostratigraphy of the Neogene Huaitoutala section in the eastern Qaidam Basin on the NE Tibetan Plateau, Qinghai Province, China and its implication on tectonic uplift of the NE Tibetan Plateau. Earth Planet. Sci. Lett. 258: 293–306.

[bib23] Fay J. C. Wu C.-I., 2000 Hitchhiking under positive Darwinian selection. Genetics 155: 1405–1413. 10880498 10.1093/genetics/155.3.1405 PMC1461156

[bib24] Fogle H., 1977 Self-pollination and its implications in peach improvement. Fruit Var. J. 31: 74–75.

[bib25] Fogle H. W. Dermen H., 1969 Genetic and chimeral constitution of three leaf variegations in the peach. J. Hered. 60: 323–328.

[bib26] Fresnedo-Ramírez J. Martínez-García P. J. Parfitt D. E. Crisosto C. H. Gradziel T. M., 2013 Heterogeneity in the entire genome for three genotypes of peach [*Prunus persica* (L.) Batsch] as distinguished from sequence analysis of genomic variants. BMC Genomics 14: 750. 24182359 10.1186/1471-2164-14-750 PMC4046826

[bib27] Fumagalli M. Vieira F. G. Linderoth T. Nielsen R., 2014 *ngsTools*: methods for population genetics analyses from next-generation sequencing data. Bioinformatics 30: 1486–1487. 24458950 10.1093/bioinformatics/btu041 PMC4016704

[bib28] Gaut B. S. Díez C. M. Morrell P. L., 2015 Genomics and the contrasting dynamics of annual and perennial domestication. Trends Genet. 31: 709–719. 26603610 10.1016/j.tig.2015.10.002

[bib29] Glémin S. Bazin E. Charlesworth D., 2006 Impact of mating systems on patterns of sequence polymorphism in flowering plants. Proc. Biol. Sci. 273: 3011–3019. 17015349 10.1098/rspb.2006.3657 PMC1639510

[bib30] Gradziel T. M., 2011 Origin and dissemination of almond. Hortic. Rev. (Am. Soc. Hortic. Sci.) 38: 23–81.

[bib31] Gross B. L. Henk A. D. Richards C. M. Fazio G. Volk G. M., 2014 Genetic diversity in *Malus* × domestica (Rosaceae) through time in response to domestication. Am. J. Bot. 101: 1770–1779. 25326619 10.3732/ajb.1400297

[bib32] Hamrick J. L. Godt M. J. W. Sherman-Broyles S. L., 1992 Factors influencing levels of genetic diversity in woody plant species, pp. 95–124 in Population Genetics of Forest Trees. Springer, New York.

[bib33] Hanada T. Watari A. Kibe T. Yamane H. Wünsch Blanco A., 2014 Two novel self-compatible *S* haplotypes in peach (*Prunus persica*). J. Jpn. Soc. Hortic. Sci. 83: 203–213.

[bib34] Hazzouri K. M. Escobar J. S. Ness R. W. Killian Newman L. Randle A. M., 2013 Comparative population genomics in *Collinsia* sister species reveals evidence for reduced effective population size, relaxed selection, and evolution of biased gene conversion with an ongoing mating system shift. Evolution 67: 1263–1278. 23617907 10.1111/evo.12027

[bib35] Hedrick U. P. Howe G. H. Taylor O. M. Tubergen C. B., 1917 The Peaches of New York. JB Lyon Company, Albany, NY.

[bib36] Holsinger K. E. Weir B. S., 2009 Genetics in geographically structured populations: defining, estimating and interpreting FST. Nat. Rev. Genet. 10: 639–650. 19687804 10.1038/nrg2611 PMC4687486

[bib37] Jo Y. Chu H. Cho J. K. Choi H. Lian S., 2015 *De novo* transcriptome assembly of two different peach cultivars grown in Korea. Genom. Data 6: 260–261. 26697390 10.1016/j.gdata.2015.10.014 PMC4664764

[bib38] Kester D. E. Sartori E., 1966 Rooting of cuttings in populations of peach (*Prunus persica* l.), almond (*Prunus amygdalus* batsch) and their F1 hybrids. Proc. Am. Soc. Hortic. Sci. 88: 219–223.

[bib39] Koepke T. Schaeffer S. Harper A. Dicenta F. Edwards M., 2013 Comparative genomics analysis in Prunoideae to identify biologically relevant polymorphisms. Plant Biotechnol. J. 11: 883–893. 23763653 10.1111/pbi.12081 PMC3775899

[bib40] Korneliussen T. S. Albrechtsen A. Nielsen R., 2014 ANGSD: analysis of next generation sequencing data. BMC Bioinformatics 15: 356. 25420514 10.1186/s12859-014-0356-4 PMC4248462

[bib41] Ladizinsky G., 1999 On the origin of almond. Genet. Resour. Crop Evol. 46: 143–147.

[bib42] Li, H., 2013 Aligning sequence reads, clone sequences and assembly contigs with BWA-MEM. arXiv: 1303.3997v1 [q-bio.GN].

[bib43] Li X.-w. Meng X.-q. Jia H.-j. Yu M.-l. Ma R.-j., 2013 Peach genetic resources: diversity, population structure and linkage disequilibrium. BMC Genet. 14: 1. 24041442 10.1186/1471-2156-14-84 PMC3848491

[bib44] Martínez-Gómez P. Arulsekar S. Potter D. Gradziel T. M., 2003 An extended interspecific gene pool available to peach and almond breeding as characterized using simple sequence repeat (SSR) markers. Euphytica 131: 313–322.

[bib45] McKey D. Elias M. Pujol B. Duputié A., 2010 The evolutionary ecology of clonally propagated domesticated plants. New Phytol. 186: 318–332. 20202131 10.1111/j.1469-8137.2010.03210.x

[bib46] Meyer R. S. DuVal A. E. Jensen H. R., 2012 Patterns and processes in crop domestication: an historical review and quantitative analysis of 203 global food crops. New Phytol. 196: 29–48. 22889076 10.1111/j.1469-8137.2012.04253.x

[bib47] Micheletti D. Dettori M. T. Micali S. Aramini V. Pacheco I., 2015 Whole-genome analysis of diversity and SNP-major gene association in peach germplasm. PLoS One 10: e0136803. 26352671 10.1371/journal.pone.0136803 PMC4564248

[bib48] Miller A. J. Gross B. L., 2011 From forest to field: perennial fruit crop domestication. Am. J. Bot. 98: 1389–1414. 21865506 10.3732/ajb.1000522

[bib49] Miller P. J. Parfitt D. E. Weinbaum S. A., 1989 Outcrossing in peach. HortScience 24: 359–360.

[bib50] Molnar P. Boos W. R. Battisti D. S., 2010 Orographic controls on climate and paleoclimate of Asia: thermal and mechanical roles for the Tibetan Plateau. Annu. Rev. Earth Planet. Sci. 38: 77.

[bib51] Mousavi S. Alisoltani A. Shiran B. Fallahi H. Ebrahimie E., 2014 *De novo* transcriptome assembly and comparative analysis of differentially expressed genes in *Prunus dulcis* Mill. in response to freezing stress. PLoS One 9: e104541. 25122458 10.1371/journal.pone.0104541 PMC4133227

[bib52] Mowrey B. D. Werner D. J. Byrne D. H., 1990 Isozyme survey of various species of *Prunus* in the subgenus *Amygdalus*. Sci. Hortic. (Amsterdam) 44: 251–260.

[bib53] Myles S. Boyko A. R. Owens C. L. Brown P. J. Grassi F., 2011 Genetic structure and domestication history of the grape. Proc. Natl. Acad. Sci. USA 108: 3530–3535. 21245334 10.1073/pnas.1009363108 PMC3048109

[bib54] Nei M. Li W.-H., 1979 Mathematical model for studying genetic variation in terms of restriction endonucleases. Proc. Natl. Acad. Sci. USA 76: 5269–5273. 291943 10.1073/pnas.76.10.5269 PMC413122

[bib55] Okie W. R., 1998 Handbook of Peach and Nectarine Varieties. Performance in the Southeastern United States and Index of Names. Agriculture Handbook, Washington.

[bib56] Potter D., 2011 Prunus, pp. 129–145 in Wild Crop Relatives: Genomic and Breeding Resources. Springer, New York.

[bib57] Rehder A., 1940 Manual of Cultivated Trees and Shrubs. Macmillan Company, New York.

[bib58] Ross-Ibarra J. Morrell P. L. Gaut B. S., 2007 Plant domestication, a unique opportunity to identify the genetic basis of adaptation. Proc. Natl. Acad. Sci. USA 104: 8641–8648. 17494757 10.1073/pnas.0700643104 PMC1876441

[bib59] Sanhueza D. Vizoso P. Balic I. Campos-Vargas R. Meneses C., 2015 Transcriptomic analysis of fruit stored under cold conditions using controlled atmosphere in *Prunus persica* cv. “Red Pearl.” Front. Plant Sci. 6: 788. 26483806 10.3389/fpls.2015.00788 PMC4586424

[bib60] Schiffels S. Durbin R., 2014 Inferring human population size and separation history from multiple genome sequences. Nat. Genet. 46: 919–925. 24952747 10.1038/ng.3015 PMC4116295

[bib61] Scorza R. Okie W. R., 1991 Peaches (*Prunus*). Acta Hortic. (290): 177–234.

[bib62] Shen Z. Ma R. Cai Z. Yu M. Zhang Z., 2015 Diversity, population structure, and evolution of local peach cultivars in china identified by simple sequence repeats. Genet. Mol. Res. 14: 101. 25729941 10.4238/2015.January.15.13

[bib63] Skotte L. Korneliussen T. S. Albrechtsen A., 2013 Estimating individual admixture proportions from next generation sequencing data. Genetics 195: 693–702. 24026093 10.1534/genetics.113.154138 PMC3813857

[bib64] Slotte T. Hazzouri K. M. Ågren J. A. Koenig D. Maumus F., 2013 The *Capsella rubella* genome and the genomic consequences of rapid mating system evolution. Nat. Genet. 45: 831–835. 23749190 10.1038/ng.2669

[bib65] Spiegel-Roy P., 1986 Domestication of fruit trees. Dev. Agric. Managed-For. Ecol. 16: 201–211.

[bib66] Su T. Wilf P. Huang Y. Zhang S. Zhou Z., 2015 Peaches preceded humans: fossil evidence from SW China. Sci. Rep. 5: 16794. 26610240 10.1038/srep16794 PMC4660870

[bib67] Tajima F., 1989 Statistical method for testing the neutral mutation hypothesis by DNA polymorphism. Genetics 123: 585–595. 2513255 10.1093/genetics/123.3.585 PMC1203831

[bib68] Tao R. Watari A. Hanada T. Habu T. Yaegaki H., 2007 Self-compatible peach (*Prunus persica*) has mutant versions of the *S* haplotypes found in self-incompatible *Prunus* species. Plant Mol. Biol. 63: 109–123. 17006593 10.1007/s11103-006-9076-0

[bib69] Verde I. Abbott A. G. Scalabrin S. Jung S. Shu S., 2013 The high-quality draft genome of peach (*Prunus persica*) identifies unique patterns of genetic diversity, domestication and genome evolution. Nat. Genet. 45: 487–494. 23525075 10.1038/ng.2586

[bib70] Vieira J. Fonseca N. A. Santos R. A. Habu T. Tao R., 2008 The number, age, sharing and relatedness of *S*-locus specificities in *Prunus*. Genet. Res. 90: 17–26. 10.1017/S0016672307009044 18289397

[bib71] Wang J. Street N. R. Scofield D. G. Ingvarsson P. K., 2016 Natural selection and recombination rate variation shape nucleotide polymorphism across the genomes of three related *Populus* species. Genetics 202: 1185–1200. 26721855 10.1534/genetics.115.183152 PMC4788117

[bib72] Wang L. Zhao S. Gu C. Zhou Y. Zhou H., 2013 Deep RNA-seq uncovers the peach transcriptome landscape. Plant Mol. Biol. 83: 365–377. 23783411 10.1007/s11103-013-0093-5

[bib73] Wellington R. Stout A. B. Einset O. Van Alstyne L. M., 1929 Pollination of fruit trees. Bulletin of the New York State Agricultural Experiment Station 577: 3–54.

[bib74] Wright S. I. Kalisz S. Slotte T., 2013 Evolutionary consequences of self-fertilization in plants. Proc. R. Soc. Lond. B Biol. Sci. 280: 20130133. 10.1098/rspb.2013.0133 PMC3652455 23595268

[bib75] Wu J. Gu C. Khan M. A. Wu J. Gao Y., 2013 Molecular determinants and mechanisms of gametophytic self-incompatibility in fruit trees of Rosaceae. Crit. Rev. Plant Sci. 32: 53–68.

[bib76] Zeinalabedini M. Khayam-Nekoui M. Grigorian V. Gradziel T. Martinez-Gomez P., 2010 The origin and dissemination of the cultivated almond as determined by nuclear and chloroplast SSR marker analysis. Sci. Hortic. (Amsterdam) 125: 593–601.

[bib77] Zeng K. Fu Y.-X. Shi S. Wu C.-I., 2006 Statistical tests for detecting positive selection by utilizing high-frequency variants. Genetics 174: 1431–1439. 16951063 10.1534/genetics.106.061432 PMC1667063

[bib78] Zheng Y. Crawford G. W. Chen X., 2014 Archaeological evidence for peach (*Prunus persica*) cultivation and domestication in China. PLoS One 9: e106595. 25192436 10.1371/journal.pone.0106595 PMC4156326

[bib79] Zohary D. Hopf M. Weiss E., 2012 Domestication of Plants in the Old World. Oxford University Press, Oxford.

