# Supplementary material for: Evolutionary Genomics of Peach and Almond Domestication
Source: G3 (Bethesda). 2016 Oct 4;6(12):3985–93. doi: 10.1534/g3.116.032672 (PMC5144968; doi:10.1534/g3.116.032672)
Supplement: Supplemental Material [file supp_6_12_3985__index.html]

Evolutionary Genomics of Peach and Almond Domestication — Supplemental Material 

# Evolutionary Genomics of Peach and Almond Domestication

## Supplemental Material for Velasco, *et al*, 2016

**Files in this Data Supplement:**

- File S1 - This file contains all supplemental tables and figures. (.pdf, 2.01 MB)
- Table S1 - Detailed sample information for *P. dulcis*, *P. persica*, and related species used in analyses. (.pdf, 199 KB)
- Table S2 - RNA-seq data used in expression analyses. (.pdf, 173 KB)
- Table S3 - Inbreeding values of peach and almond samples. (.pdf, 178 KB)
- Table S4 - Mean F*ST*, diversity statistics, and neutrality test values. (.pdf, 198 KB)
- Table S5 - Significant GO terms for *FST* candidate genes based on top 5% quantile. (.pdf, 318 KB)
- Table S6 - Number and mean summary statistic values of non-genic and genic windows (NGW and GW, respectively) in the lowest 5% quantile for Tajima?s D, Zeng?s E, Fay &Wu?s H, and θπ for each species. (.pdf, 184 KB)
- Table S7 - Mann-Whitney U (MWU) and X2 tests for significance of RNAseq specificity and tissue specific expression of peach fruit, peach leaf, almond ovary, or almond anther and candidate status. (.pdf, 198 KB)
- Figure S1 - Peach and almond fruit and seed anatomy. (.tiff, 4.77 MB)
- Figure S2 - Mean mapped depth of peach and almond sequences used in this analysis filtered for mapping quality (MAPQ) scores 30 and base quality scores 20. (.tiff, 24 KB)
- Figure S3 - Distribution of inbreeding values for almond and peach samples studied. (.pdf, 24 KB)
- Figure S4 - Increasing the assumed clusters to *K*=6 (right) places PD01, the almond-peach F1 hybrid collected from Kharkiv Market, Ukraine, into a unique sub-population. (.tiff, 3.08 MB)
- Figure S5 - Nucleotide diversity (θπ) in almond for each chromosome. The vertical red line indicates the approximate location of the centromere. (.tiff, 762 KB)
- Figure S6 - Nucleotide diversity (θπ) in peach for each chromosome. (.tiff, 661 KB)
- Figure S7 - *FST* between almond and peach for each chromosome. (.tiff, 754 KB)
- Figure S8 - Historical changes in N*e* over time in both almond and peach. (.tiff, 146 KB)
