# Supplementary material for: Evolutionary Genomics of Peach and Almond Domestication
Source: G3 (Bethesda). 2016 Oct 4;6(12):3985–93. doi: 10.1534/g3.116.032672 (PMC5144968; doi:10.1534/g3.116.032672)
Supplement: Supplemental Material [file supp_g3.116.032672_FileS1.pdf]

Table S1 Detailed sample information for *P. dulcis*, *P. persica*, and related species used in analyses.

Species	Sample ID	Accession and/or Cultivar	Avg. Depth	Origin	Source	Ref
P. dulcis	PD01	DPRU 2578.2, #53	30.46	Ukraine	SRR4036105[w]	1[y]
	PD02	Tardy Nonpareil	34.59	USA	SRR4036108[x]	1[y]
	PD03	DPRU 1791.3, BE-1609	17.93	Turkey	SRR4045225[w]	1[z]
	PD04	DPRU 2374.12	16.77	Iran	SRR4045227[w]	1[z]
	PD05	DPRU 1456.4, Badam	15.90	Pakistan	SRR4045228[w]	1[z]
	PD06	DPRU 2301, Tuono	17.23	Italy	SRR4045226[w]	1[z]
	PD07	DPRU 1462.2	19.38	Pakistan	SRR4045229[w]	1[z]
	PD08	DPRU 1207.2	14.47	Uzbekistan	SRR4045222[w]	1[z]
	PD09	DPRU 2331.9	17.17	China	SRR4045224[w]	1[z]
	PD10	DPRU 0210, Languedoc	20.63	France	SRR4045223[w]	1[z]
	PD11	S3067	6.64	Spain	SRR765861	2
	PD12	D05-187	4.72	Spain	SRR765850	2
	PD13	Lauranne	13.00	France	SRR765838	2
	PD14	Ramillete	6.69	Spain	SRR765679	2
P. persica	PP02	Yumyeong	22.37	Korea	SRR502994	3
	PP03	Shenzhou Mitao	11.19	N China	SRR502993, SRR502992	3
	PP04	Sahua Hong Pantao	14.46	S China	SRR502991, SRR502990	3
	PP05	Quetta	12.64	Pakistan	SRR502989, SRR502987	3
	PP06	Oro A	25.78	Brazil	SRR502986	3
	PP07	IF7310828	12.75	Italy	SRR503001, SRR503000	3
	PP08	GF305	18.68	France	SRR502983	3
	PP09	F_1 Contender × Ambra	15.57	Italy	SRR502997	3
	PP10	Earligold	35.40	USA	SRR502996, SRR502995	3
	PP11	Bolero	22.42	Italy	SRR501836	3
	PP12	F8,1-42	11.88	USA	SRR068361	4
	PP13	Georgia Belle	13.13	USA	SRR068359	4
	PP14	Dr. Davis	14.44	USA	SRR068360	4
	PP15	Lovell	37.36	USA	SRR40365107[x]	1[y]
P. cerasifera (outgroup)	PC01	DPRU 0579, Myrobalan	35.02	USA	SRR40365106[w]	1[y]

Source: newly resequenced samples provided by [w]United States Department of Agriculture National Clonal Germplasm Repository (Davis) or [x]University of California, Davis; **Reference:** [1]this study (resequencing of samples performed at [y]BGI or [z]UC Berkeley), [2]Koepke *et al.* 2013, [3]Verde *et al.* 2013, [4]Ahmad *et al.* 2011

Table S2 RNA-seq data used in expression analyses.

SRA Run ID	Species	Tissue	Cultivar	Reference
SRR2086434	Peach	fruit mesocarp	Red Pearl	Sanhueza *et al.* 2015
SRR2086433	Peach	fruit mesocarp	Red Pearl	Sanhueza *et al.* 2015
SRR3823906	Peach	fruit mesocarp	DU-88	N/A
SRR3823907	Peach	fruit mesocarp	DU-88	N/A
SRR2290949	Peach	leaf	Jangtaek	Jo *et al.* 2015
SRR2290951	Peach	leaf	Mibaek	Jo *et al.* 2015
SRR1662173	Peach	leaf	Hongyetao	Wang *et al.* 2013
SRR1662174	Peach	leaf	Mantianhong	Wang *et al.* 2013
SRR2976060	Almond	ovary	genotype H	Mousavi *et al.* 2014
SRR2976058	Almond	anther	genotype H	Mousavi *et al.* 2014

N/A: unable to locate a publication for this data submitted to SRA by the Andres Bello Universidad

Table S3 Inbreeding values of peach and almond samples.

**Peach**	*F*	**Almond**	*F*
PP02	0.072	PD02	0.000
PP03	0.222	PD03	0.002
PP04	0.116	PD04	0.000
PP05	0.001	PD05	0.002
PP06	0.533	PD06	0.000
PP07	0.081	PD07	0.000
PP08	0.737	PD08	0.000
PP09	0.000	PD09	0.000
PP10	0.064	PD10	0.000
PP11	0.000	PD11	0.000
PP13	0.000	PD12	0.027
PP14	0.176	PD13	0.000
PP15	0.557	PD14	0.000
Mean	*0.197*	*Mean*	*0.002*

Table S4 Mean F_{ST}, diversity statistics, and neutrality test values.

		Almond				Peach			
**Region**	**F_{ST}**	$\theta_\pi \times 10^3$	***D***	***H***	***E***	$\theta_\pi \times 10^3$	***D***	***H***	***E***
genome	0.586	18.374	-1.150	-0.115	-0.223	2.700	-0.492	-0.561	0.139
genic	0.606	10.570	-1.489	-0.030	-0.351	1.667	-0.510	-0.497	0.101
non-genic	0.568	25.668	-0.834	-0.195	-0.103	3.611	-0.476	-0.617	0.173
Chr 1	0.605	16.706	-1.266	-0.154	-0.231	2.022	-0.559	-0.513	0.096
Chr 2	0.557	20.222	-1.094	-0.081	-0.227	4.014	-0.462	-0.579	0.158
Chr 3	0.593	16.858	-1.130	-0.116	-0.217	2.455	-0.417	-0.557	0.155
Chr 4	0.558	21.779	-0.994	-0.110	-0.187	3.707	-0.326	-0.565	0.186
Chr 5	0.589	17.602	-1.184	-0.092	-0.243	2.352	-0.544	-0.593	0.139
Chr 6	0.611	16.042	-1.177	-0.125	-0.225	2.121	-0.512	-0.533	0.119
Chr 7	0.586	18.793	-1.166	-0.105	-0.232	2.613	-0.461	-0.575	0.154
Chr 8	0.575	19.972	-1.119	-0.097	-0.225	2.593	-0.651	-0.635	0.137

Table S5 Significant GO terms for F_{ST} candidate genes based on top 5% quantile. (Type: F - molecular function; P - biological process)

GO acc	Type	Term	Query Item	BG Item	p-value	FDR
GO:0030554	F	adenyl nucleotide binding	153	2225	7.7e-07	9.3e-05
GO:0005524	F	ATP binding	146	2104	8.8e-07	9.3e-05
GO:0005515	F	protein binding	122	1634	2.5e-07	9.3e-05
GO:0001883	F	purine nucleoside binding	153	2225	7.7e-07	9.3e-05
GO:0001882	F	nucleoside binding	153	2226	7.9e-07	9.3e-05
GO:0032559	F	adenyl ribonucleotide binding	146	2104	8.8e-07	9.3e-05
GO:0017076	F	purine nucleotide binding	162	2437	2.3e-06	0.00017
GO:0016772	F	transferase activity, transferring phosphorus-containing groups	101	1347	2.4e-06	0.00017
GO:0032555	F	purine ribonucleotide binding	155	2313	2.6e-06	0.00017
GO:0032553	F	ribonucleotide binding	155	2313	2.6e-06	0.00017
GO:0000166	F	nucleotide binding	165	2531	5.4e-06	0.00031
GO:0004713	F	protein tyrosine kinase activity	62	751	1.7e-05	0.00077
GO:0016798	F	hydrolase activity, acting on glycosyl bonds	37	363	1.6e-05	0.00077
GO:0004553	F	hydrolase activity, hydrolyzing O-glycosyl compounds	36	348	1.6e-05	0.00077
GO:0004888	F	transmembrane receptor activity	26	213	1.9e-05	0.00082
GO:0004872	F	receptor activity	26	215	2.2e-05	0.00089
GO:0060089	F	molecular transducer activity	31	285	2.5e-05	0.0009
GO:0004871	F	signal transducer activity	31	285	2.5e-05	0.0009
GO:0016740	F	transferase activity	137	2103	3.7e-05	0.0013
GO:0003964	F	RNA-directed DNA polymerase activity	16	101	5.1e-05	0.0016
GO:0034061	F	DNA polymerase activity	17	115	6.5e-05	0.002
GO:0016265	P	death	38	377	1.6e-05	0.0042
GO:0012501	P	programmed cell death	36	357	2.6e-05	0.0042
GO:0023052	P	signaling	45	486	2e-05	0.0042
GO:0008219	P	cell death	38	377	1.6e-05	0.0042
GO:0006915	P	apoptosis	36	357	2.6e-05	0.0042
GO:0006278	P	RNA-dependent DNA replication	16	101	5.1e-05	0.0068
GO:0016773	F	phosphotransferase activity, alcohol group as acceptor	75	1072	0.00035	0.0097
GO:0003824	F	catalytic activity	344	6355	0.00034	0.0097
GO:0016779	F	nucleotidyltransferase activity	23	217	0.00037	0.01
GO:0004672	F	protein kinase activity	70	989	0.0004	0.01
GO:0006260	P	DNA replication	19	150	0.00016	0.01
GO:0002376	P	immune system process	20	165	0.00018	0.015
GO:0006955	P	immune response	20	165	0.00018	0.015
GO:0045087	P	innate immune response	20	165	0.00018	0.015
GO:0016301	F	kinase activity	74	1089	0.00082	0.02
GO:0023046	P	signaling process	36	409	0.00032	0.021

Continued on next page

GO acc	Type	Term	Query Item	BG Item	p-value	FDR
GO:0023060	P	signal transmission	36	409	0.00032	0.021
GO:0005488	F	binding	371	7025	0.0011	0.025
GO:0005215	F	transporter activity	50	682	0.0013	0.03
GO:0007165	P	signal transduction	33	378	0.00063	0.039
GO:0007154	P	cell communication	14	107	0.00083	0.048

■ **Table S6** Number and mean summary statistic values of non-genic and genic windows (NGW and GW, respectively) in the lowest 5% quantile for Tajima's D, Zeng's E, Fay & Wu's H, and θ_π for each species. The same information is shown for windows in the top and bottom 5% quantiles for F_{ST}. Also included are the number of genes represented by genic windows and the ratio of genic to non-genic windows.

**Statistic**		NGW	Mean	GW	Genes	Mean	GW:NGW
Tajima's D	almond	17112	-2.3015	203826	10365	-2.3302	11.9113
	peach	126724	-2.0946	93781	6000	-2.0870	0.7400
Zeng's E	almond	12969	-0.6501	195992	11385	-0.6606	15.1123
	peach	81258	-0.5535	129763	10706	-0.5494	1.5969
Fay & Wu's H	almond	127429	-1.0246	38095	4029	-1.0325	0.2990
	peach	107582	-2.9458	105573	8526	-3.0076	0.9813
θ_π	almond	13360	0.0033	188322	9647	0.0035	14.0960
	peach	58287	8.6123e-06	124818	9927	7.3075e-06	2.1414
F_{ST}	top 5%	73406	0.8716	88596	7400	0.8587	1.2069
	bottom 5%	51018	0.1739	35688	4692	0.1622	0.6995

Table S7 Mann-Whitney U (MWU) and X^2 tests for significance of RNAseq specificity and tissue specific expression of peach fruit, peach leaf, almond ovary, or almond anther and candidate status.

			Almond	Peach
**Tissue**	**Test**	$\mathbf{F}_{ST}$	E	E
**Fruit** (peach)	χ^2	0.1284	0.0052	0.0530
	MWU	0.0423	0.1109	0.0987
**Leaf** (peach)	χ^2	1.362×10^{-9}	1.52×10^{-15}	6.779×10^{-4}
	MWU	3.653×10^{-4}	0.1642	0.1216
**Ovary** (almond)	χ^2	0.909	0.0150	0.0074
	MWU	0.6004	0.3680	0.6040
**Anther** (almond)	χ^2	0.7448	0.0014	0.0921
	MWU	0.4083	0.6753	0.5490

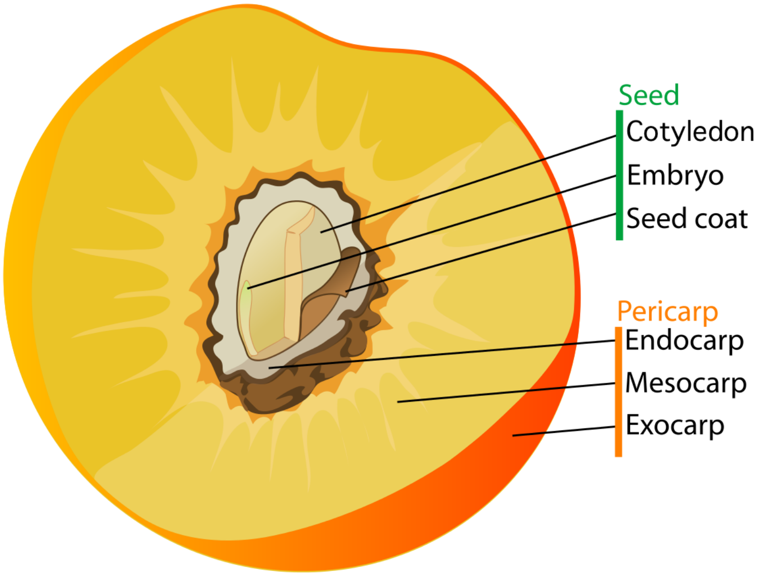

Figure S1 Peach and almond fruit and seed anatomy.

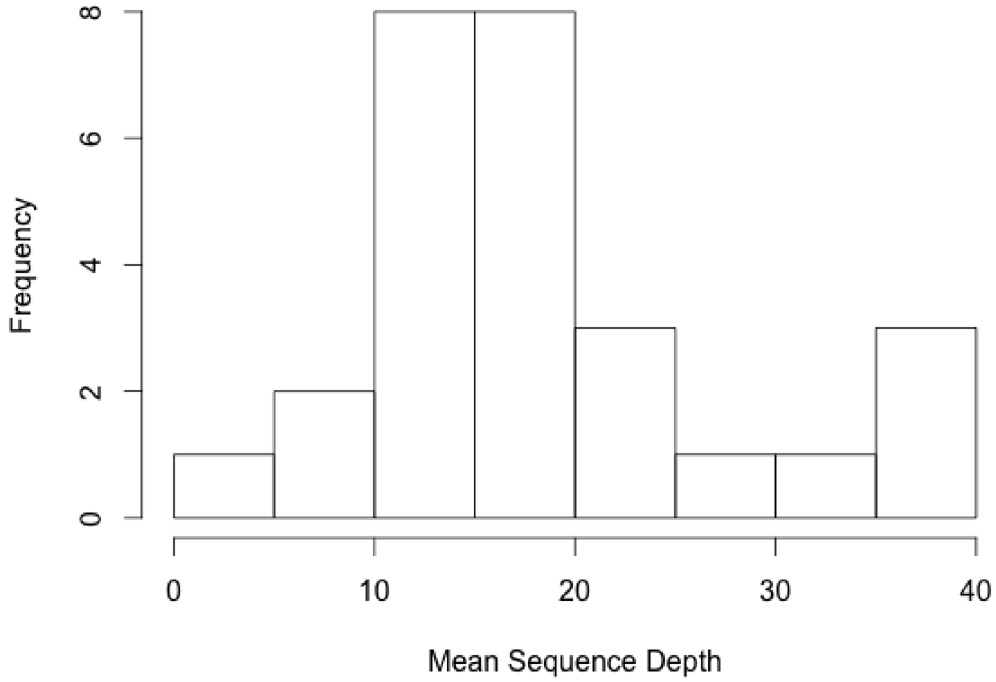

Figure S2 Mean mapped depth of peach and almond sequences used in this analysis filtered for mapping quality (MAPQ) scores ≥ 30 and base quality scores ≥ 20.

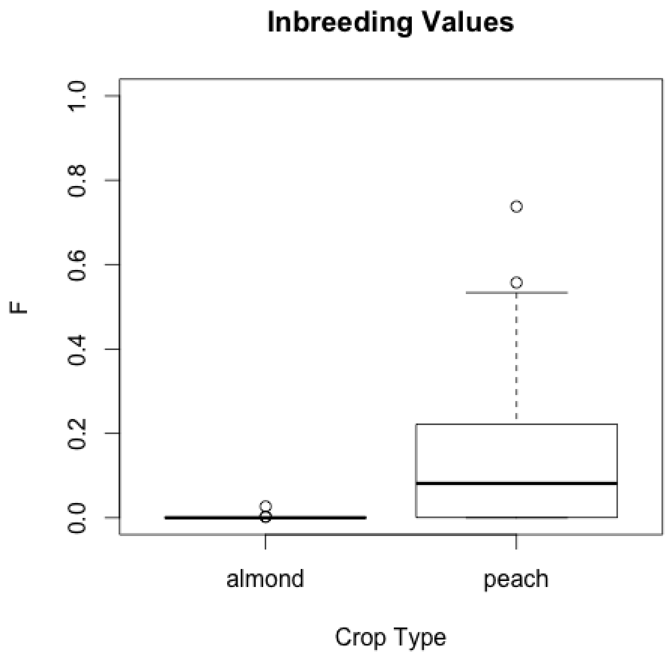

Figure S3 Distribution of inbreeding values for almond and peach samples studied.

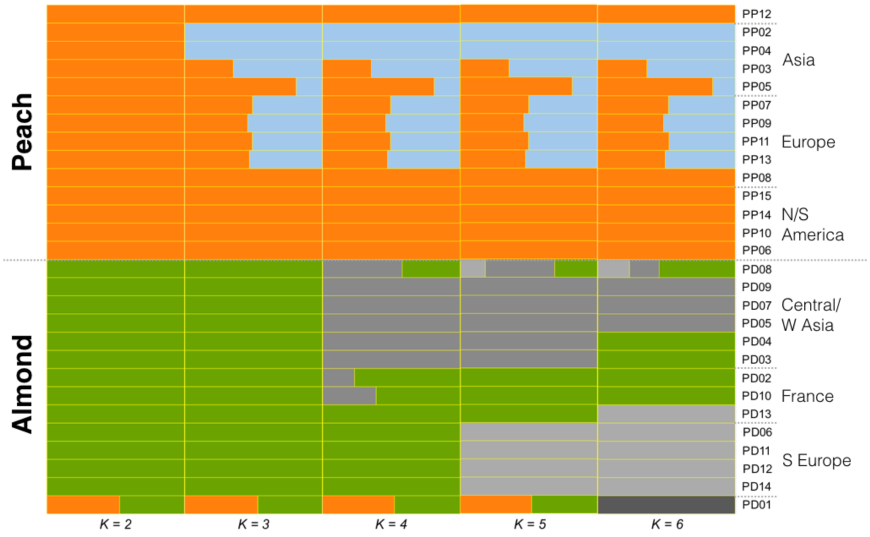

Figure S4 Increasing the assumed clusters to K=6 (right) places PD01, the almond-peach F_1 hybrid collected from Kharkiv Market, Ukraine, into a unique sub-population. It also shifts the assignments of samples PD13, PD03, and PD04 to different sub-populations, when compared to their assignments in K=5 (second from right).

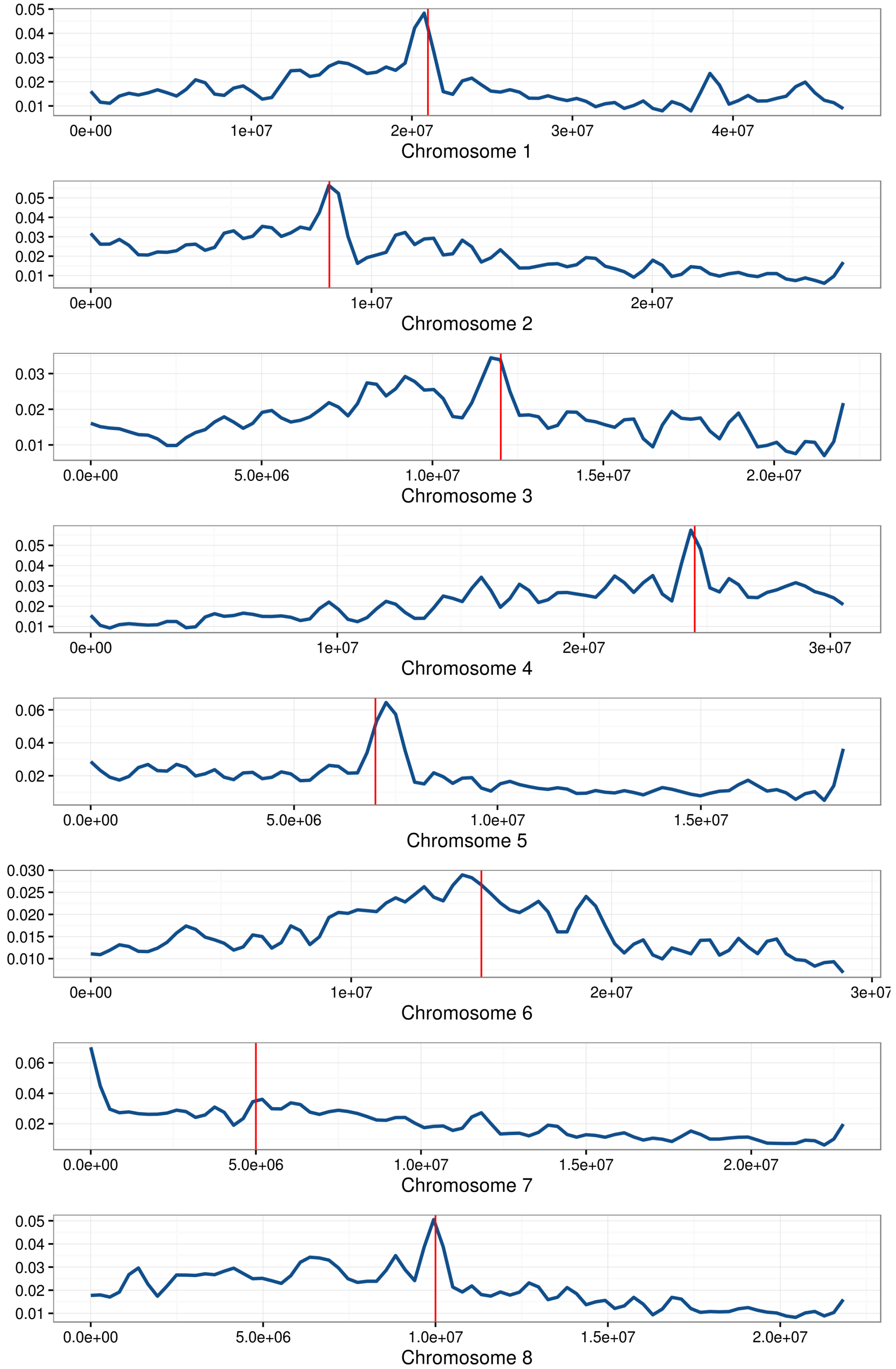

Figure S5 Nucleotide diversity (θ_π) in almond for each chromosome. The vertical red line indicates the approximate location of the centromere.

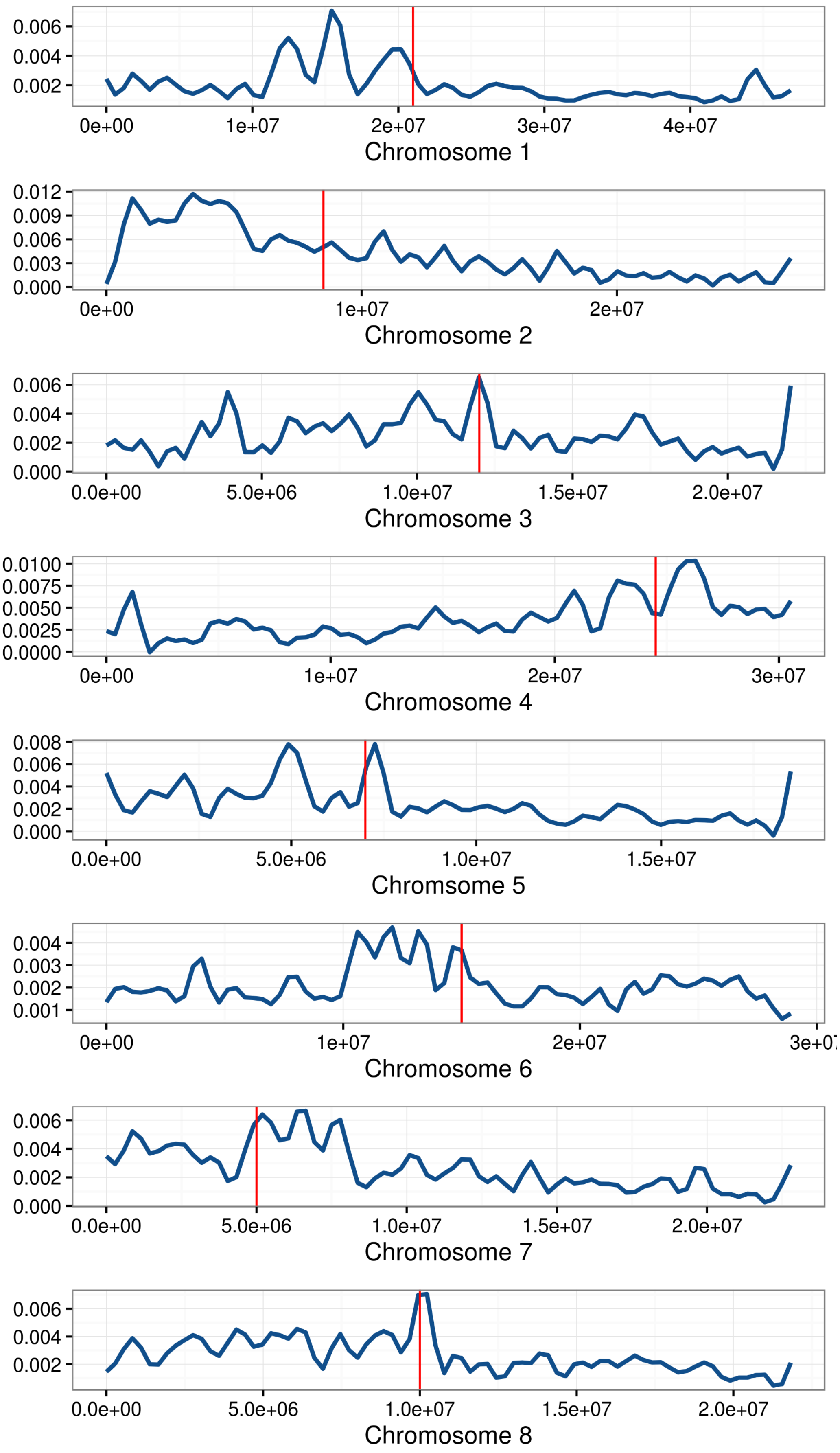

Figure S6 Nucleotide diversity (θ_π) in peach for each chromosome. The vertical red line indicates the approximate location of the centromere.

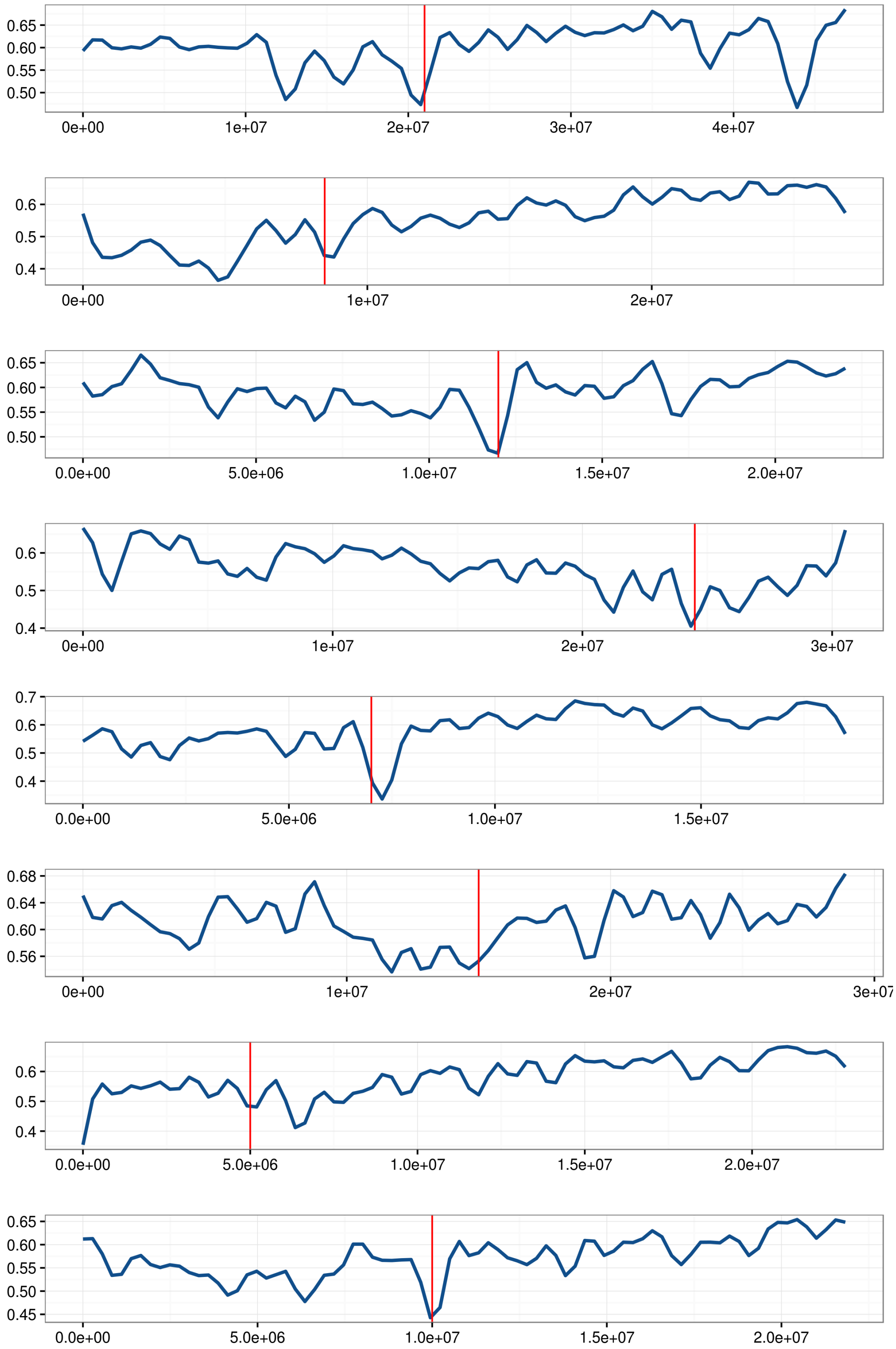

Figure S7 F_{ST} between almond and peach for each chromosome. The vertical red line indicates the approximate location of the centromere.

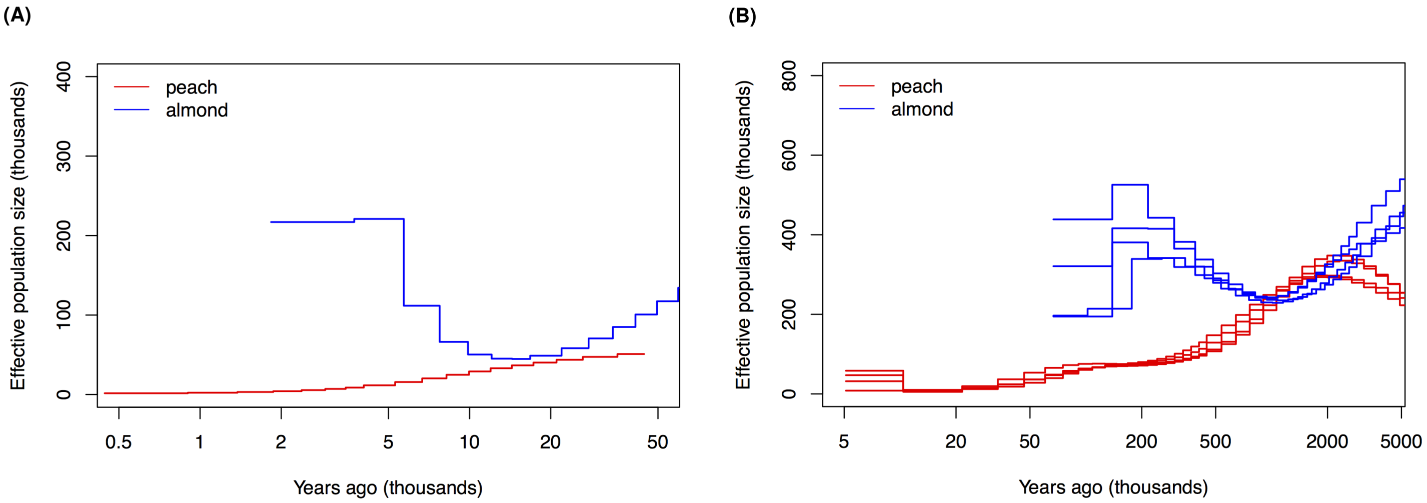

Figure S8 Historical changes in N_e over time in both almond and peach. Shown are estimates of N_e for both the (A) recent ($\leq 50 \times 10^3$ BP) and (B) ancient ($\leq 5000 \times 10^3$ BP) past.
