# Supplementary material for: Evolutionary Genomics of Peach and Almond Domestication
Source: G3 (Bethesda). 2016 Oct 4;6(12):3985–93. doi: 10.1534/g3.116.032672 (PMC5144968; doi:10.1534/g3.116.032672)
Supplement: Supplemental Material [file supp_g3.116.032672_TableS1.pdf]

Table S1 Detailed sample information for *P. dulcis*, *P. persica*, and related species used in analyses.

Species	Sample ID	Accession and/or Cultivar	Avg. Depth	Origin	Source	Ref
P. dulcis	PD01	DPRU 2578.2, #53	30.46	Ukraine	SRR4036105[w]	1[y]
	PD02	Tardy Nonpareil	34.59	USA	SRR4036108[x]	1[y]
	PD03	DPRU 1791.3, BE-1609	17.93	Turkey	SRR4045225[w]	1[z]
	PD04	DPRU 2374.12	16.77	Iran	SRR4045227[w]	1[z]
	PD05	DPRU 1456.4, Badam	15.90	Pakistan	SRR4045228[w]	1[z]
	PD06	DPRU 2301, Tuono	17.23	Italy	SRR4045226[w]	1[z]
	PD07	DPRU 1462.2	19.38	Pakistan	SRR4045229[w]	1[z]
	PD08	DPRU 1207.2	14.47	Uzbekistan	SRR4045222[w]	1[z]
	PD09	DPRU 2331.9	17.17	China	SRR4045224[w]	1[z]
	PD10	DPRU 0210, Languedoc	20.63	France	SRR4045223[w]	1[z]
	PD11	S3067	6.64	Spain	SRR765861	2
	PD12	D05-187	4.72	Spain	SRR765850	2
	PD13	Lauranne	13.00	France	SRR765838	2
	PD14	Ramillete	6.69	Spain	SRR765679	2
P. persica	PP02	Yumyeong	22.37	Korea	SRR502994	3
	PP03	Shenzhou Mitao	11.19	N China	SRR502993, SRR502992	3
	PP04	Sahua Hong Pantao	14.46	S China	SRR502991, SRR502990	3
	PP05	Quetta	12.64	Pakistan	SRR502989, SRR502987	3
	PP06	Oro A	25.78	Brazil	SRR502986	3
	PP07	IF7310828	12.75	Italy	SRR503001, SRR503000	3
	PP08	GF305	18.68	France	SRR502983	3
	PP09	F_1 Contender × Ambra	15.57	Italy	SRR502997	3
	PP10	Earligold	35.40	USA	SRR502996, SRR502995	3
	PP11	Bolero	22.42	Italy	SRR501836	3
	PP12	F8,1-42	11.88	USA	SRR068361	4
	PP13	Georgia Belle	13.13	USA	SRR068359	4
	PP14	Dr. Davis	14.44	USA	SRR068360	4
	PP15	Lovell	37.36	USA	SRR40365107[x]	1[y]
P. cerasifera (outgroup)	PC01	DPRU 0579, Myrobalan	35.02	USA	SRR40365106[w]	1[y]

Source: newly resequenced samples provided by [w]United States Department of Agriculture National Clonal Germplasm Repository (Davis) or [x]University of California, Davis; **Reference:** [1]this study (resequencing of samples performed at [y]BGI or [z]UC Berkeley), [2]Koepke *et al.* 2013, [3]Verde *et al.* 2013, [4]Ahmad *et al.* 2011
