# Supplementary material for: Evolutionary Genomics of Peach and Almond Domestication
Source: G3 (Bethesda). 2016 Oct 4;6(12):3985–93. doi: 10.1534/g3.116.032672 (PMC5144968; doi:10.1534/g3.116.032672)
Supplement: Supplemental Material [file supp_g3.116.032672_TableS2.pdf]

Table S2 RNA-seq data used in expression analyses.

SRA Run ID	Species	Tissue	Cultivar	Reference
SRR2086434	Peach	fruit mesocarp	Red Pearl	Sanhueza *et al.* 2015
SRR2086433	Peach	fruit mesocarp	Red Pearl	Sanhueza *et al.* 2015
SRR3823906	Peach	fruit mesocarp	DU-88	N/A
SRR3823907	Peach	fruit mesocarp	DU-88	N/A
SRR2290949	Peach	leaf	Jangtaek	Jo *et al.* 2015
SRR2290951	Peach	leaf	Mibaek	Jo *et al.* 2015
SRR1662173	Peach	leaf	Hongyetao	Wang *et al.* 2013
SRR1662174	Peach	leaf	Mantianhong	Wang *et al.* 2013
SRR2976060	Almond	ovary	genotype H	Mousavi *et al.* 2014
SRR2976058	Almond	anther	genotype H	Mousavi *et al.* 2014

N/A: unable to locate a publication for this data submitted to SRA by the Andres Bello Universidad
