# Supplementary material for: Evolutionary Genomics of Peach and Almond Domestication
Source: G3 (Bethesda). 2016 Oct 4;6(12):3985–93. doi: 10.1534/g3.116.032672 (PMC5144968; doi:10.1534/g3.116.032672)
Supplement: Supplemental Material [file supp_g3.116.032672_TableS3.pdf]

■ **Table S3** Inbreeding values of peach and almond samples.

**Peach**	*F*	**Almond**	*F*
PP02	0.072	PD02	0.000
PP03	0.222	PD03	0.002
PP04	0.116	PD04	0.000
PP05	0.001	PD05	0.002
PP06	0.533	PD06	0.000
PP07	0.081	PD07	0.000
PP08	0.737	PD08	0.000
PP09	0.000	PD09	0.000
PP10	0.064	PD10	0.000
PP11	0.000	PD11	0.000
PP13	0.000	PD12	0.027
PP14	0.176	PD13	0.000
PP15	0.557	PD14	0.000
Mean	*0.197*	*Mean*	*0.002*
