# Supplementary material for: Evolutionary Genomics of Peach and Almond Domestication
Source: G3 (Bethesda). 2016 Oct 4;6(12):3985–93. doi: 10.1534/g3.116.032672 (PMC5144968; doi:10.1534/g3.116.032672)
Supplement: Supplemental Material [file supp_g3.116.032672_TableS4.pdf]

Table S4 Mean F_{ST}, diversity statistics, and neutrality test values.

		Almond				Peach			
**Region**	$\mathbf{F}_{ST}$	$\theta_\pi \times 10^3$	***D***	***H***	***E***	$\theta_\pi \times 10^3$	***D***	***H***	***E***
genome	0.586	18.374	-1.150	-0.115	-0.223	2.700	-0.492	-0.561	0.139
genic	0.606	10.570	-1.489	-0.030	-0.351	1.667	-0.510	-0.497	0.101
non-genic	0.568	25.668	-0.834	-0.195	-0.103	3.611	-0.476	-0.617	0.173
Chr 1	0.605	16.706	-1.266	-0.154	-0.231	2.022	-0.559	-0.513	0.096
Chr 2	0.557	20.222	-1.094	-0.081	-0.227	4.014	-0.462	-0.579	0.158
Chr 3	0.593	16.858	-1.130	-0.116	-0.217	2.455	-0.417	-0.557	0.155
Chr 4	0.558	21.779	-0.994	-0.110	-0.187	3.707	-0.326	-0.565	0.186
Chr 5	0.589	17.602	-1.184	-0.092	-0.243	2.352	-0.544	-0.593	0.139
Chr 6	0.611	16.042	-1.177	-0.125	-0.225	2.121	-0.512	-0.533	0.119
Chr 7	0.586	18.793	-1.166	-0.105	-0.232	2.613	-0.461	-0.575	0.154
Chr 8	0.575	19.972	-1.119	-0.097	-0.225	2.593	-0.651	-0.635	0.137
