# Supplementary material for: Evolutionary Genomics of Peach and Almond Domestication
Source: G3 (Bethesda). 2016 Oct 4;6(12):3985–93. doi: 10.1534/g3.116.032672 (PMC5144968; doi:10.1534/g3.116.032672)
Supplement: Supplemental Material [file supp_g3.116.032672_TableS5.pdf]

Table S5 Significant GO terms for F_{ST} candidate genes based on top 5% quantile. (Type: F - molecular function; P - biological process)

GO acc	Type	Term	Query Item	BG Item	p-value	FDR
GO:0030554	F	adenyl nucleotide binding	153	2225	7.7e-07	9.3e-05
GO:0005524	F	ATP binding	146	2104	8.8e-07	9.3e-05
GO:0005515	F	protein binding	122	1634	2.5e-07	9.3e-05
GO:0001883	F	purine nucleoside binding	153	2225	7.7e-07	9.3e-05
GO:0001882	F	nucleoside binding	153	2226	7.9e-07	9.3e-05
GO:0032559	F	adenyl ribonucleotide binding	146	2104	8.8e-07	9.3e-05
GO:0017076	F	purine nucleotide binding	162	2437	2.3e-06	0.00017
GO:0016772	F	transferase activity, transferring phosphorus-containing groups	101	1347	2.4e-06	0.00017
GO:0032555	F	purine ribonucleotide binding	155	2313	2.6e-06	0.00017
GO:0032553	F	ribonucleotide binding	155	2313	2.6e-06	0.00017
GO:0000166	F	nucleotide binding	165	2531	5.4e-06	0.00031
GO:0004713	F	protein tyrosine kinase activity	62	751	1.7e-05	0.00077
GO:0016798	F	hydrolase activity, acting on glycosyl bonds	37	363	1.6e-05	0.00077
GO:0004553	F	hydrolase activity, hydrolyzing O-glycosyl compounds	36	348	1.6e-05	0.00077
GO:0004888	F	transmembrane receptor activity	26	213	1.9e-05	0.00082
GO:0004872	F	receptor activity	26	215	2.2e-05	0.00089
GO:0060089	F	molecular transducer activity	31	285	2.5e-05	0.0009
GO:0004871	F	signal transducer activity	31	285	2.5e-05	0.0009
GO:0016740	F	transferase activity	137	2103	3.7e-05	0.0013
GO:0003964	F	RNA-directed DNA polymerase activity	16	101	5.1e-05	0.0016
GO:0034061	F	DNA polymerase activity	17	115	6.5e-05	0.002
GO:0016265	P	death	38	377	1.6e-05	0.0042
GO:0012501	P	programmed cell death	36	357	2.6e-05	0.0042
GO:0023052	P	signaling	45	486	2e-05	0.0042
GO:0008219	P	cell death	38	377	1.6e-05	0.0042
GO:0006915	P	apoptosis	36	357	2.6e-05	0.0042
GO:0006278	P	RNA-dependent DNA replication	16	101	5.1e-05	0.0068
GO:0016773	F	phosphotransferase activity, alcohol group as acceptor	75	1072	0.00035	0.0097
GO:0003824	F	catalytic activity	344	6355	0.00034	0.0097
GO:0016779	F	nucleotidyltransferase activity	23	217	0.00037	0.01
GO:0004672	F	protein kinase activity	70	989	0.0004	0.01
GO:0006260	P	DNA replication	19	150	0.00016	0.01
GO:0002376	P	immune system process	20	165	0.00018	0.015
GO:0006955	P	immune response	20	165	0.00018	0.015
GO:0045087	P	innate immune response	20	165	0.00018	0.015
GO:0016301	F	kinase activity	74	1089	0.00082	0.02
GO:0023046	P	signaling process	36	409	0.00032	0.021

Continued on next page

GO acc	Type	Term	Query Item	BG Item	p-value	FDR
GO:0023060	P	signal transmission	36	409	0.00032	0.021
GO:0005488	F	binding	371	7025	0.0011	0.025
GO:0005215	F	transporter activity	50	682	0.0013	0.03
GO:0007165	P	signal transduction	33	378	0.00063	0.039
GO:0007154	P	cell communication	14	107	0.00083	0.048
