# Supplementary material for: Evolutionary Genomics of Peach and Almond Domestication
Source: G3 (Bethesda). 2016 Oct 4;6(12):3985–93. doi: 10.1534/g3.116.032672 (PMC5144968; doi:10.1534/g3.116.032672)
Supplement: Supplemental Material [file supp_g3.116.032672_TableS6.pdf]

Table S6 Number and mean summary statistic values of non-genic and genic windows (NGW and GW, respectively) in the lowest 5% quantile for Tajima's D, Zeng's E, Fay & Wu's H, and θ_π for each species. The same information is shown for windows in the top and bottom 5% quantiles for F_{ST}. Also included are the number of genes represented by genic windows and the ratio of genic to non-genic windows.

**Statistic**		NGW	Mean	GW	Genes	Mean	GW:NGW
Tajima's D	almond	17112	-2.3015	203826	10365	-2.3302	11.9113
	peach	126724	-2.0946	93781	6000	-2.0870	0.7400
Zeng's E	almond	12969	-0.6501	195992	11385	-0.6606	15.1123
	peach	81258	-0.5535	129763	10706	-0.5494	1.5969
Fay & Wu's H	almond	127429	-1.0246	38095	4029	-1.0325	0.2990
	peach	107582	-2.9458	105573	8526	-3.0076	0.9813
θ_π	almond	13360	0.0033	188322	9647	0.0035	14.0960
	peach	58287	8.6123e-06	124818	9927	7.3075e-06	2.1414
F_{ST}	top 5%	73406	0.8716	88596	7400	0.8587	1.2069
	bottom 5%	51018	0.1739	35688	4692	0.1622	0.6995
