# Supplementary material for: Evolutionary Genomics of Peach and Almond Domestication
Source: G3 (Bethesda). 2016 Oct 4;6(12):3985–93. doi: 10.1534/g3.116.032672 (PMC5144968; doi:10.1534/g3.116.032672)
Supplement: Supplemental Material [file supp_g3.116.032672_TableS7.pdf]

■ **Table S7** Mann-Whitney U (MWU) and X^2 tests for significance of RNAseq specificity and tissue specific expression of peach fruit, peach leaf, almond ovary, or almond anther and candidate status.

			**Almond**	**Peach**
**Tissue**	**Test**	$\mathbf{F}_{ST}$	***E***	***E***
**Fruit** (peach)	χ^2	0.1284	0.0052	0.0530
	MWU	0.0423	0.1109	0.0987
**Leaf** (peach)	χ^2	1.362×10^{-9}	1.52×10^{-15}	6.779×10^{-4}
	MWU	3.653×10^{-4}	0.1642	0.1216
**Ovary** (almond)	χ^2	0.909	0.0150	0.0074
	MWU	0.6004	0.3680	0.6040
**Anther** (almond)	χ^2	0.7448	0.0014	0.0921
	MWU	0.4083	0.6753	0.5490
