# Supplementary figures and images for: Evolutionary Genomics of Peach and Almond Domestication
Source: G3 (Bethesda). 2016 Oct 4;6(12):3985–93. doi: 10.1534/g3.116.032672 (PMC5144968; doi:10.1534/g3.116.032672)

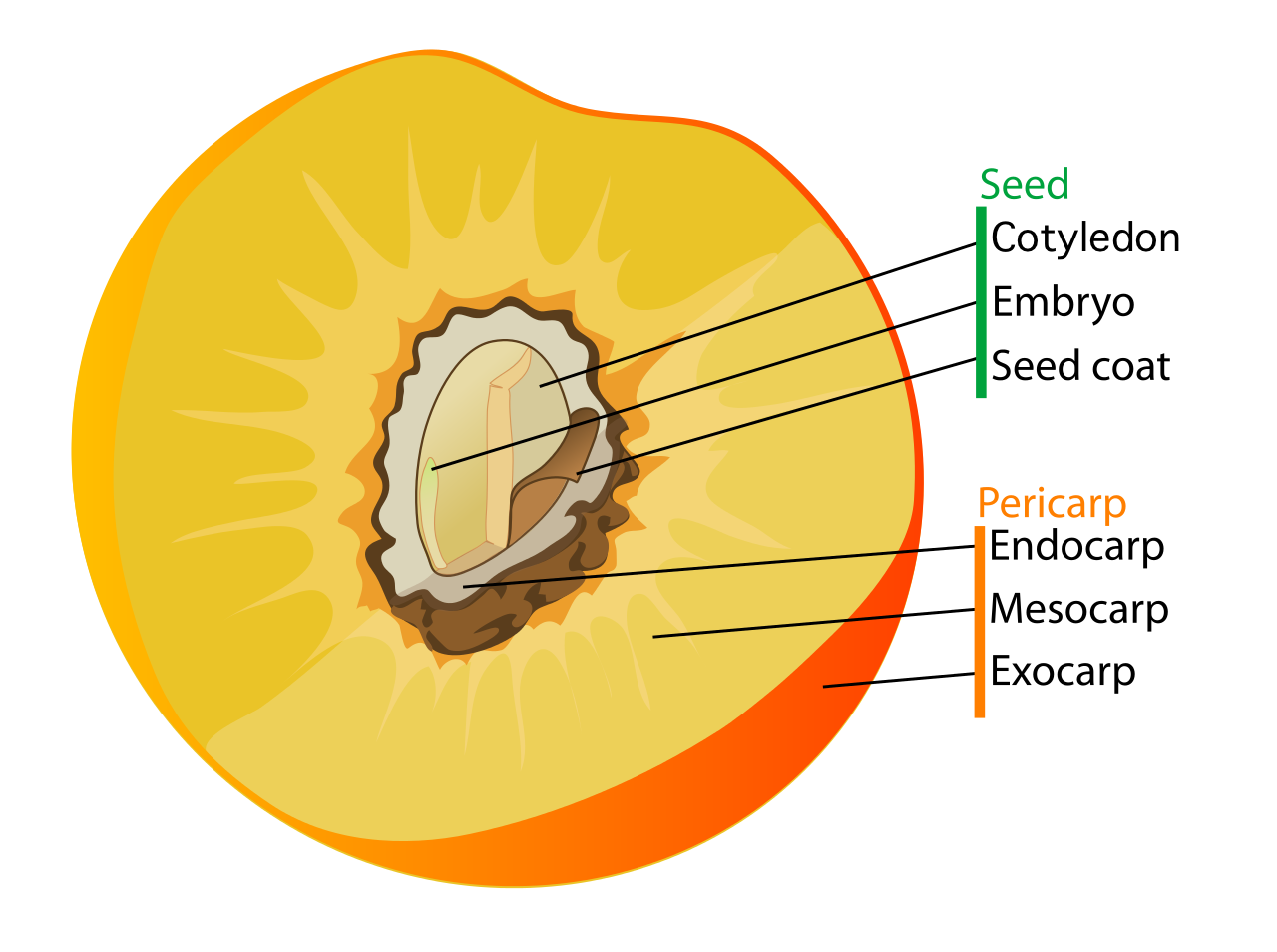

Supplement: Supplemental Material [file supp_g3.116.032672_FigureS1.tif]

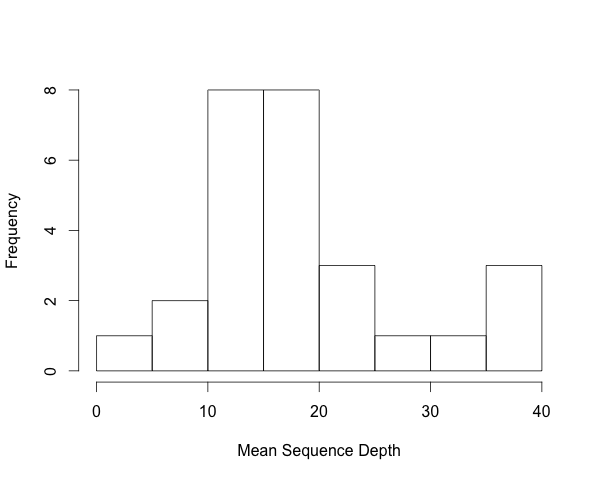

Supplement: Supplemental Material [file supp_g3.116.032672_FigureS2.tif]

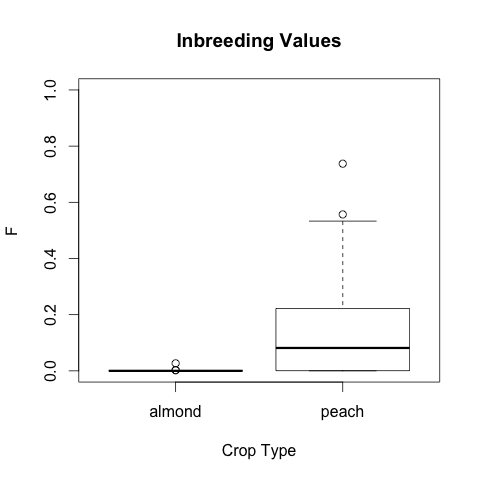

Supplement: Supplemental Material [file supp_g3.116.032672_FigureS3.tif]

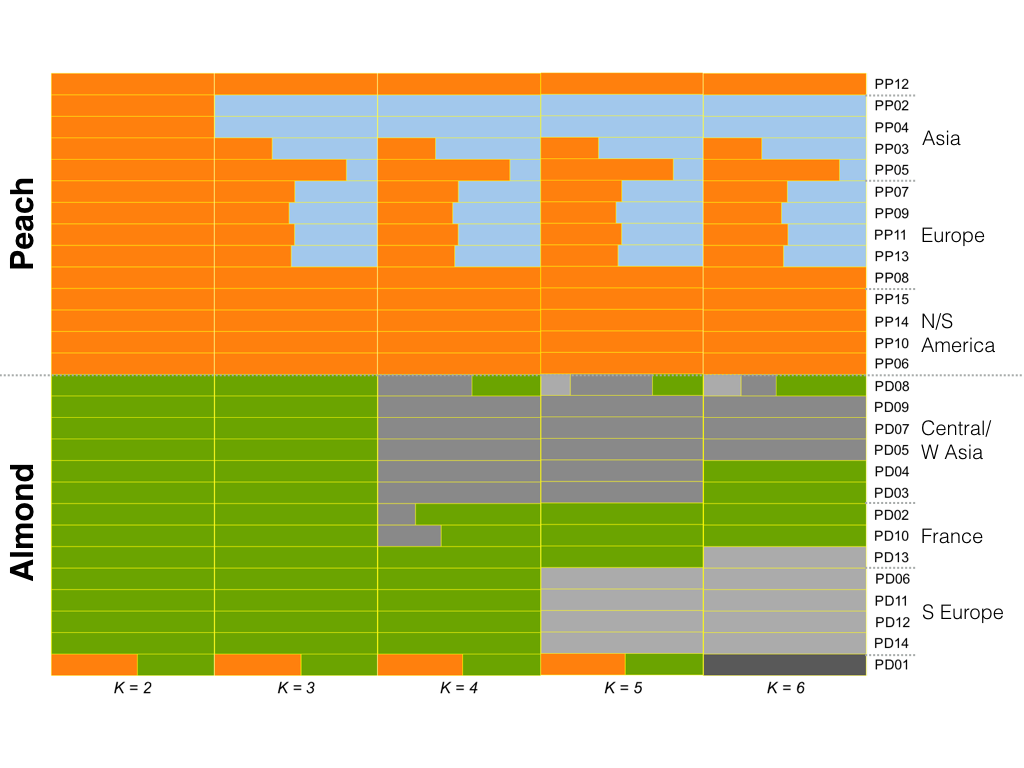

Supplement: Supplemental Material [file supp_g3.116.032672_FigureS4.tif]

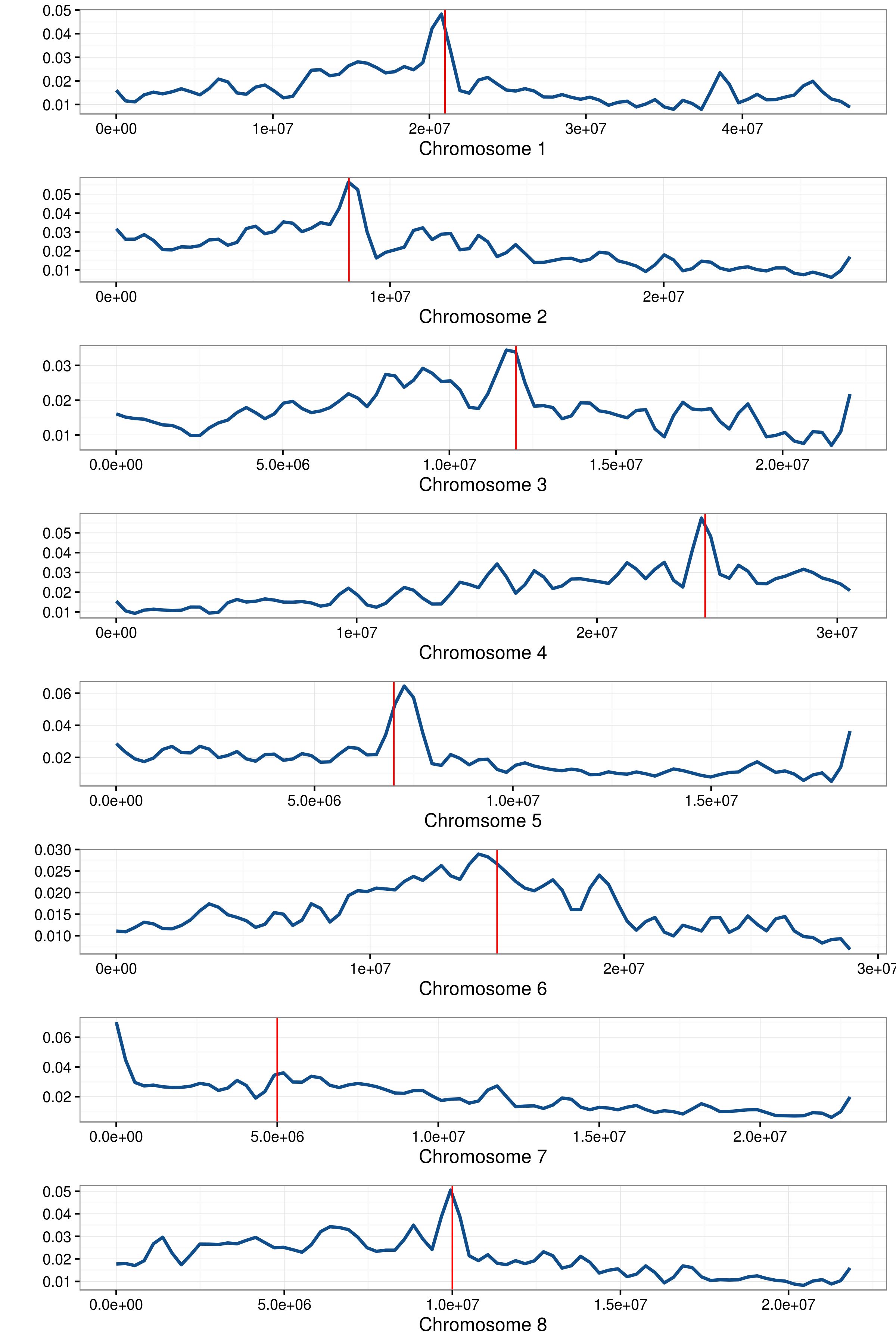

Supplement: Supplemental Material [file supp_g3.116.032672_FigureS5.tif]

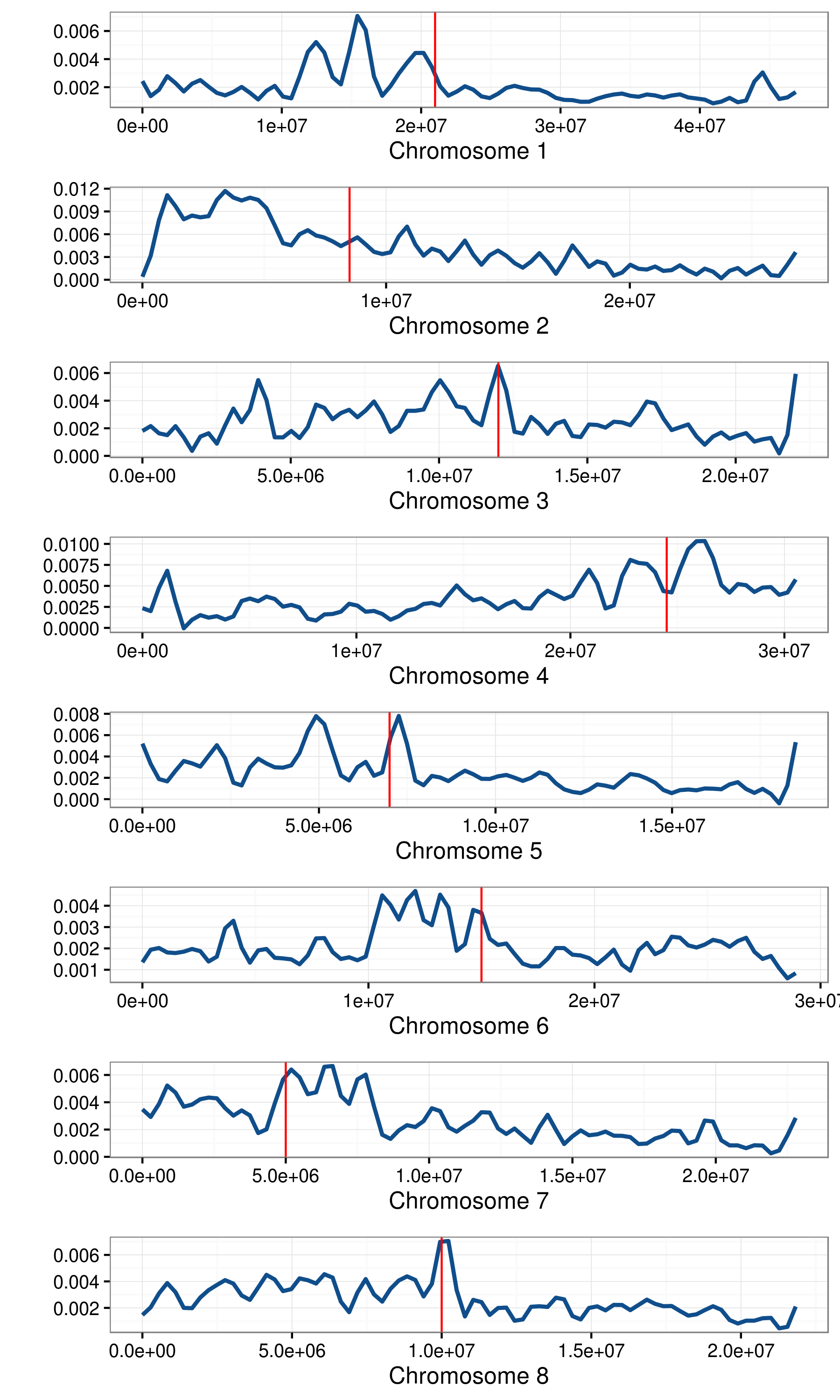

Supplement: Supplemental Material [file supp_g3.116.032672_FigureS6.tif]

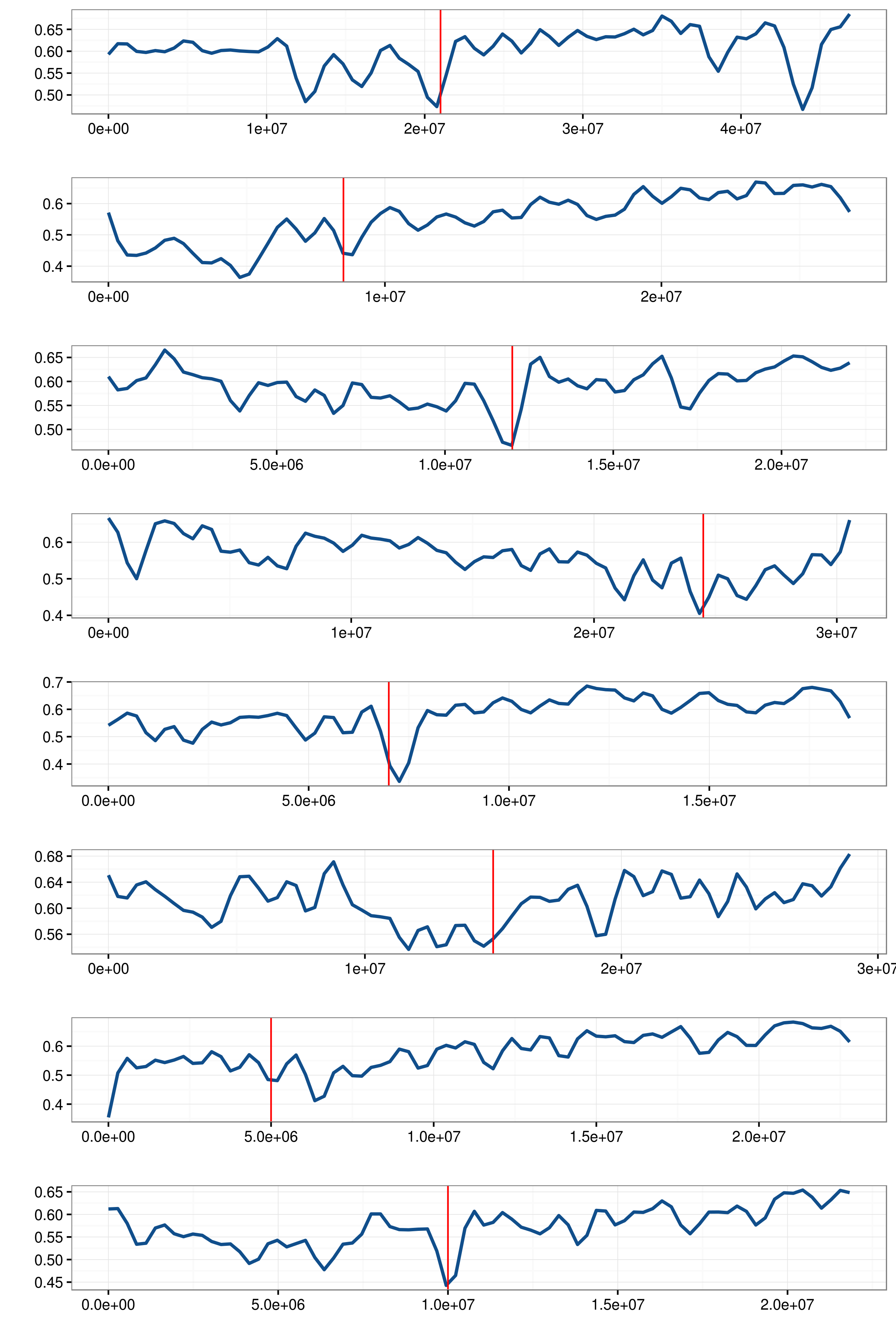

Supplement: Supplemental Material [file supp_g3.116.032672_FigureS7.tif]

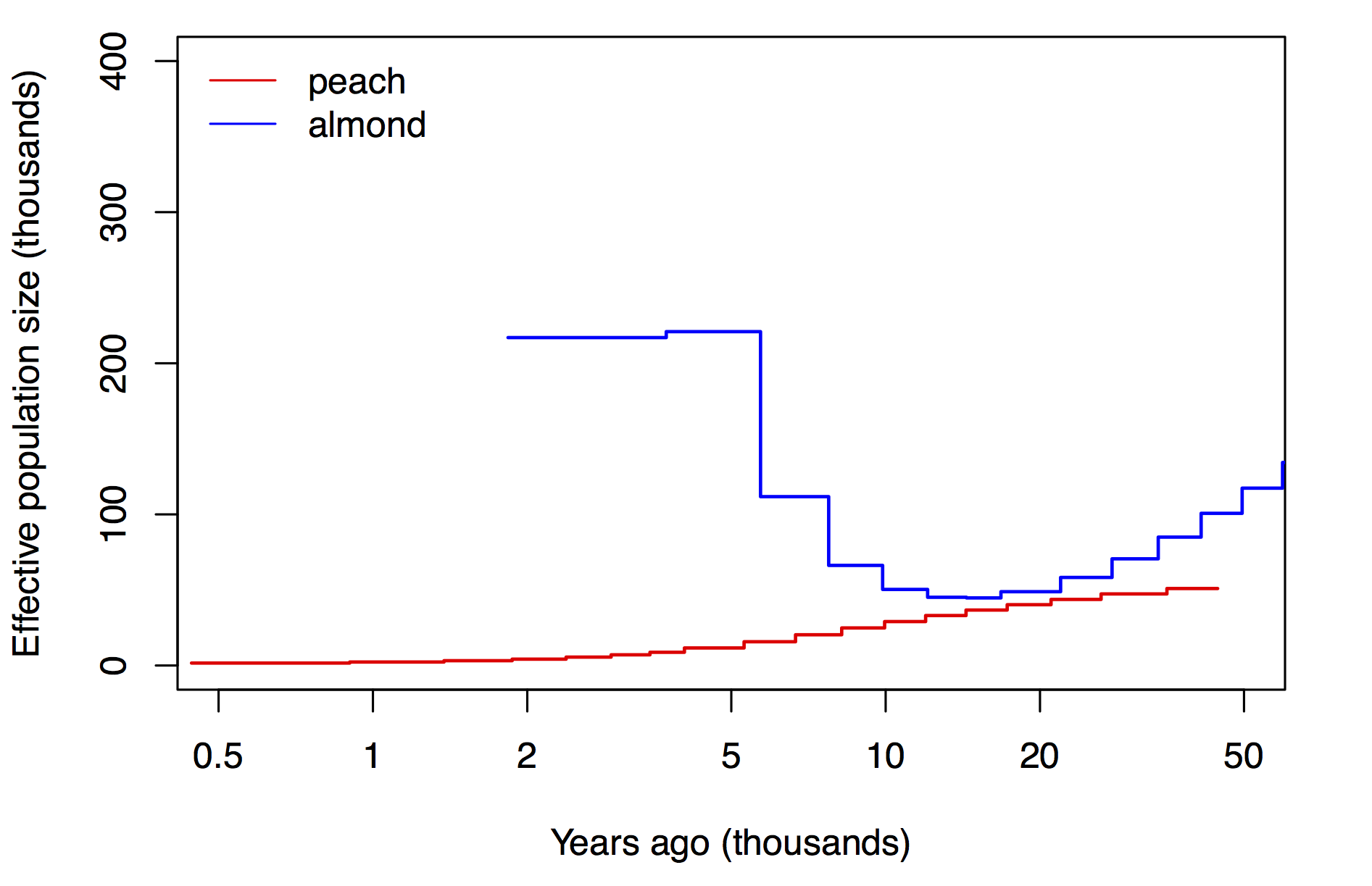

Supplement: Supplemental Material [file supp_g3.116.032672_FigureS8.tif]
